# Contrasted levels of genetic diversity in a benthic Mediterranean octocoral: Consequences of different demographic histories?

**DOI:** 10.1002/ece3.2490

**Published:** 2016-10-28

**Authors:** Moutassem Billah Masmoudi, Lamya Chaoui, Nur Eda Topçu, Pachka Hammami, Mohamed Hichem Kara, Didier Aurelle

**Affiliations:** ^1^ Aix Marseille UnivUniv AvignonCNRSIRDIMBEMarseilleFrance; ^2^ Laboratoire Bioressources MarinesUniversité d'Annaba Badji MokhtarAnnabaAlgérie; ^3^ İstanbul Üniversitesi Su Ürünleri FakültesiİstanbulTurkey; ^4^ CIRADUMR CMAEEMontpellierFrance

**Keywords:** Octocoral, Conservation Genetics, demographic history, Mediterranean Sea, *Eunicella cavolini*, microsatellite

## Abstract

Understanding the factors explaining the observed patterns of genetic diversity is an important question in evolutionary biology. We provide the first data on the genetic structure of a Mediterranean octocoral, the yellow gorgonian *Eunicella cavolini*, along with insights into the demographic history of this species. We sampled populations in four areas of the Mediterranean Sea: continental France, Algeria, Turkey, and the Balearic and Corsica islands. Along French coasts, three sites were sampled at two depths (20 and 40 m). We demonstrated a high genetic structure in this species (overall *F*_ST_ = 0.13), and most pairwise differentiation tests were significant. We did not detect any difference between depths at the same site. Clustering analyses revealed four differentiated groups corresponding to the main geographical areas. The levels of allelic richness and heterozygosity were significantly different between regions, with highest diversity in Algeria and lowest levels in Turkey. The highest levels of private allelic richness were observed in Algeria followed by Turkey. Such contrasted patterns of genetic diversity were not observed in other Mediterranean octocorals and could be the result of different evolutionary histories. We also provide new empirical evidence of contrasting results between tests and model‐based studies of demographic history. Our results have important consequences for the management of this species.

## 1 Introduction

Understanding the factors explaining the genetic diversity of species and populations is a pivotal and long‐standing question in population genetics (Ellegren & Galtier, 2016; Romiguier et al., 2014). In the context of the current global change, studying the genetic diversity of ecologically key species is important for management and conservation. Indeed genetic diversity is the fuel of an adaptive response to environmental change, and population genetics aims at estimating its distribution within and among populations. Romiguier et al. (2014) have demonstrated that life history traits, such as parental investment and fecundity, explain the main differences in diversity levels among metazoans. Inside species, differences in genetic diversity between populations can reflect varying levels of local effective size and gene flow, or particular demographic histories. For example, the latitudinal patterns of genetic diversity for terrestrial species in Europe are often determined by last glacial fluctuations (Hewitt, 2000). In the marine realm, the genetic consequences of quaternary climatic fluctuations have been studied by Maggs et al. (2008), who proposed a theoretical framework to study glacial refugia and recolonization in North Atlantic benthic species. Their predictions are based on lower levels of genetic diversity after recolonization (a pattern potentially erased by secondary contacts; Petit et al., 2003). The reconstruction of demographic history, on the basis of sequence polymorphism, also suggested demographic expansion for three benthic species following sea‐level rise in the Sunda Shelf (Crandall, Sbrocco, Deboer, Barber, & Carpenter, 2011). Nevertheless, the impact of past climatic fluctuations on the current genetic diversity remains to be studied for numerous marine species and oceanic basins.

The Mediterranean Sea is an interesting geographical and environmental context for the study of the demographic history of marine species. It comprises different basins with different current and past environmental conditions (Hayes, Kucera, Kallel, Sbaffi, & Rohling, 2005). A dozen different biogeographical areas have been described in the Mediterranean Sea which is a biodiversity hot spot (Bianchi et al., 2012). For numerous species, the different basins correspond to different genetic units (Borsa et al., 1997) which could have evolved more or less independently. The past sea‐level variation added additional constraints to marine species, with a level 120 m lower than present at the LGM around French coasts (Hayes et al., 2005; Lambeck & Bard, 2000).

Differences in levels of genetic diversity between basins have been demonstrated in several cases. Reduced levels of genetic diversity have been observed in Adriatic and Black Seas for the sprat *Sprattus sprattus* (Limborg et al., 2012), in the Eastern Mediterranean for the red gorgonian, *Paramuricea clavata* (Mokhtar‐Jamaï et al., 2011), or for deep populations of the red coral, *Corallium rubrum* (Costantini et al., 2011; but see Cannas et al., 2016). In the seagrass *Posidonia oceanica*, higher genetic diversity has been observed in central populations, potentially as the consequence of a secondary contact between Western and Eastern populations (Arnaud‐Haond et al., 2007). Different approaches allow the study of demographic history which might explain the observed differences in genetic diversity (eg, Beaumont, 1999; Cornuet & Luikart, 1996; Drummond, Rambaut, Shapiro, & Pybus, 2005; Girod, Vitalis, Leblois, & Fréville, 2011; Rogers & Harpending, 1992). In all cases, genetic structure can bias the results and should be taken into account for such approaches (Städler, Haubold, Merino, Stephan, & Pfaffelhuber, 2009).

Octocorals are good models to study patterns of genetic diversity and demographic history in the Mediterranean Sea. Previous studies have identified well‐differentiated populations for these sessile species (eg, Costantini, Fauvelot, & Abbiati, 2007b; Ledoux et al., 2010; Mokhtar‐Jamaï et al., 2011). These species present low dispersal abilities (Costantini, Fauvelot, & Abbiati, 2007a; Garrabou et al., 2009; Ledoux et al., 2010; but see Martínez‐Quintana, Bramanti, Viladrich, Rossi, & Guizien, 2015), and they could be more impacted by sea‐level and climatic fluctuations than species with higher dispersal. No clear past demographic fluctuations have been demonstrated for the red coral *Corallium rubrum* in the Mediterranean Sea (Aurelle et al., 2011; Ledoux et al., 2010) on the basis of tests of mutation–drift equilibrium, but other methods could be more informative (Girod et al., 2011).

**Figure 1:**
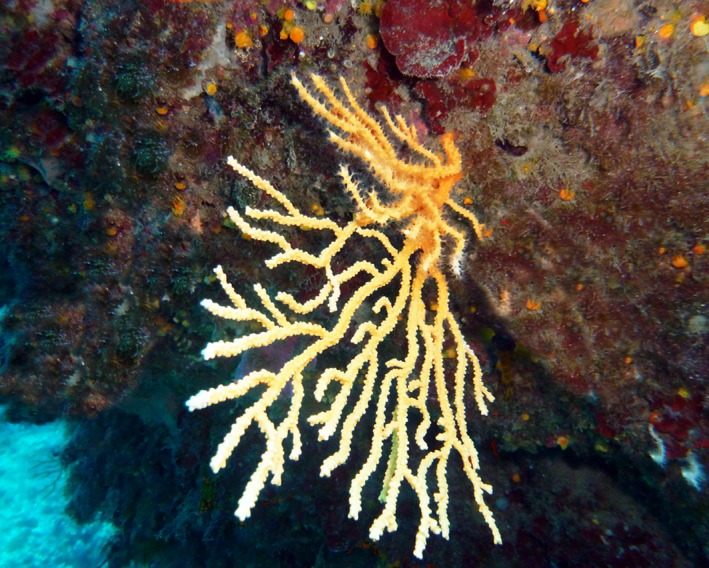
Yellow sea fan *Eunicella cavolini* of the Algerian region (Kiane, depth ~20 m). Photography credit Farid DERBAL

We studied here the genetic diversity and the genetic structure of the yellow gorgonian, *Eunicella cavolini* (Koch 1887) (Figure 1), one of the most abundant gorgonians in the Mediterranean (Weinberg, 1978). *E. cavolini* was impacted by mortality events linked with thermal anomalies over the past two decades with variable levels of necrosis depending on location, depth, and individuals (Garrabou et al., 2009). Its wide range, from Western Mediterranean to Marmara Sea, allows comparing the history of different basins. Up to now, there was no extended genetic study on this species because of a lack of adequate molecular markers (Calderon, Garrabou, & Aurelle, 2006).

Our aim was to study the genetic diversity of *E. cavolini* in different parts of the Mediterranean Sea. First, we will describe the genetic structure of this species at different spatial scales. We include a comparison between depths to test the differentiation along an environmental gradient. We will then test whether populations from different geographical areas present the same levels of diversity and similar demographic histories. We will study past demographic events with tests of mutation–drift equilibrium and with estimates of current and past effective sizes. These results will be useful for the management of this ecologically important species (Ballesteros, 2006).

## 2 Materials and methods

### 2.1 Sampling

**Figure 2:**
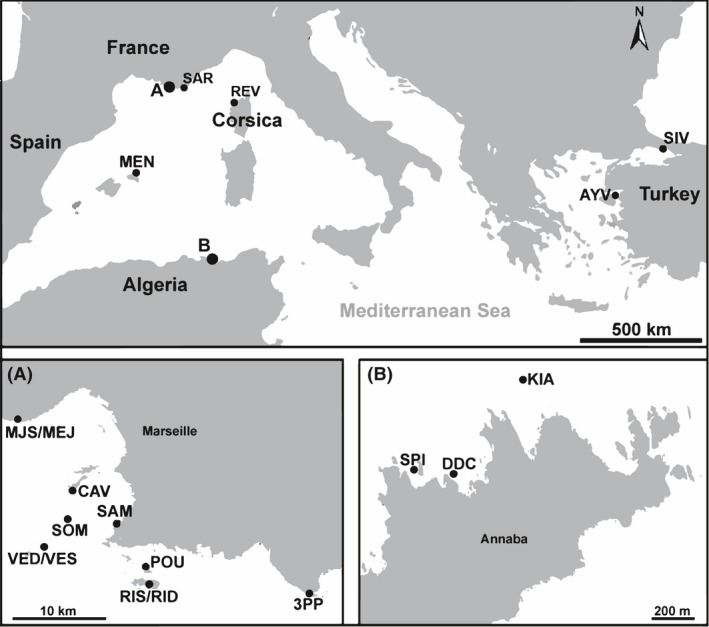
Map of the 19 *Eunicella cavolini* samples (main sites at the Mediterranean scale). (a) Samples collected at two different depths in the same location are separated by a slash in the French region of Marseille. (b) Samples from the Algerian region of Annaba (black dots)

Five hundred and eighty‐four individuals of the yellow gorgon*ian Eunicella cavolini* were sampled by scuba diving from 19 locations across the Mediterranean Sea. Several regions and sites per region were taken in consideration in order to cover most of the distribution range and to allow the study of genetic structure at distances varying from 20 m to ~2704 km. The main regions considered here were northern (Provence, Corsica, Balearic Islands) and southwestern Mediterranean (Algeria), Aegean Sea, and Marmara Sea (Figure 2). Samples collected from France included individuals collected from different depths, 20 m and 40 m, at the same sites (VED/VES, MEJ/MJS, RIS/RID) (Table 1). Small fragments (3–5 cm) were collected randomly (approximately 30 colonies sampled per site) and then preserved in 95% ethanol at −20°C for further use.

**Table 1:** Collecting sites of *Eunicella cavolini* in the Mediterranean Sea: location name, code, GPS coordinates, depth, and region, *N* = sample size, *H*_obs_ and *H*_exp_: observed and expected heterozygosities, Na: mean number of alleles per locus, [Ar(18)] and [Ap(18)]: rarefied allelic richness (for *N* = 18) and private allelic richness, *F*_IS_: fixation index, significant values are indicated in bold; *G* = number of distinct multilocus genotypes per sample, *R* = genotypic richness, *p*sex (f) = probability of the duplicate genotypes to be the result of sexual reproduction in case of duplicate MLGs

Location name	Code	GPS coordinates	Depth (m)	Region	*N*	*H*_obs_	*H*_exp_	Na	[Ar(18)]	[Ap(18)]	*F*_IS_	*G*	*R*	*p*sex (f)
Annaba—Kiane	KIA	36° 58.16′N	17–21	Algeria	30	0.62	0.70	8.14	5.44	1.13	**0.12**	30	1.00	
		7° 47.4′E												
Annaba—Espion	SPI	36° 58.118′N	18–32		30	0.50	0.61	6.86	4.65	1.05	**0.19**	30	1.00	
		7° 46.41′E												
Annaba—Dent de chien	DDC	36°57.00′N	24–27		30	0.58	0.71	8.71	5.64	0.91	**0.19**	29	0.97	5.53E‐11
		7° 42.34′E												
Menorca	MEN	40° 4′8.44″N	25	Balearic Islands	26	0.46	0.49	4.57	3.59	0.09	0.06	26	1.00	
		4° 8′31.60″E												
Revellatta	REV	42° 35.080′N	15–20	Corsica	34	0.54	0.63	6.14	4.8	0.34	**0.14**	34	1.00	
		8° 43.680′E												
Plane Island (Gulf of Lion)	POU	43° 11.340′N	15–25	France–Provence	30	0.37	0.57	4.57	4.13	0.11	**0.36**	30	1.00	
		5° 23.130′E												
Riou shallow	RIS	43° 10.360′N	20		32	0.51	0.58	6.29	4.61	0.03	**0.13**	32	1.00	
		5° 23.420′E												
Riou deep	RID	43° 10.360′N	40		31	0.51	0.58	6.14	4.53	0.09	**0.12**	31	1.00	
		5° 23.420′E												
Cap Caveau	CAV	43° 15.630′N	25		29	0.48	0.56	6.14	4.54	0.12	**0.16**	29	1.00	
		5° 17.390′E												
Veyron shallow	VES	43° 12.414′N	20		30	0.50	0.56	5.71	4.3	0.1	**0.1**	30	1.00	
		5° 15.176′E												
Veyron deep	VED	43° 12.414′N	40		30	0.51	0.63	5.71	4.46	0.09	**0.18**	30	1.00	
		5° 15.176′E												
Méjean shallow	MJS	43° 19.700′N	18–20		33	0.52	0.54	6.43	4.41	0.14	0.05	33	1.00	
		5° 13.480′E												
Méjean deep	MEJ	43° 19.700′N	30–40		31	0.47	0.54	6.00	4.53	0.15	**0.12**	31	1.00	
		5° 13.480′E												
Somlit	SOM	43° 14.050′N	58		31	0.47	0.54	5.00	3.89	0.04	**0.12**	30	0.97	0.006
		5° 17.050′E												
Saména	SAM	43° 13.780′N	10		34	0.44	0.50	4.43	3.66	0.06	**0.13**	33	0.97	0.002
		5° 20.880′E												
La Ciotat—3PP Cave	3PP	43° 9.795′N	15		30	0.34	0.54	4.57	3.66	0.09	**0.38**	30	1.00	
		5° 36.000′E												
Porquerolles –	SAR	42° 59.272′N	40		32	0.47	0.53	5.86	4.42	0.06	**0.11**	32	1.00	
Sec de Sarraniers		6° 17.503′E												
Ayvalık	AYV	39° 33.541′N	30–34	Turkey–Aegean Sea	30	0.43	0.44	3.43	2.84	0.64	0	25	0.83	4.59E‐08;2.25E‐12; 0.0003;0.0003; 0.0002
		26° 586′E												
Sivriada	SYV	40°52′26.15″N	34–38	Turkey–Marmara Sea	30	0.32	0.34	3.00	2.45	0.39	0.07	29	0.97	2.79 E‐06
		28°58′14.30″E												

### 2.2 Molecular markers

Total genomic DNA was extracted using two methods: either the QIAamp^®^ DNA Mini Kit (Qiagen) following the manufacturer's instructions or a salting‐out procedure (Mokhtar‐Jamaï et al., 2011). All individuals were genotyped at seven microsatellite loci: C21, C30, C40, S14 (Molecular Ecology Resources Primer Development Consortium et al., 2010), Ever007, Ever009 (Holland, Dawson, Horsburgh, Krupa, & Stevens, 2013), Mic56 (This study). All loci were amplified according to the PCR protocols described in Appendix S1. PCR products were analyzed on an ABI 3130 Genetic Analyser using an internal size standard (GeneScan 600 LIZ; Life Technologies). GeneMapper v.4.0 software (Applied Biosystems) was used to score alleles. We estimated null allele frequencies and tested the presence of large allele dropout using MICRO‐CHECKER v.2.2.3 (Van Oosterhout, Hutchinson, Wills, & Shipley, 2004). GENCLONE 2.0 (Arnaud‐Haond & Belkhir, 2007) was used to calculate the number of distinct multilocus genotypes (MLG) per sample (G). This resulted in the identification of nine duplicated MLGs (Appendix S2, Table S1). For the following analyses, only one representative of each MLG was retained corresponding to a final set of 575 samples.

### 2.3 Genetic diversity and tests of panmixia

The genetic diversity was analyzed using GENETIX v.4.05 (Belkhir, Borsa, Chikhi, Raufaste, & Bonhomme, 1996), with observed (*H*_obs_) and unbiased expected heterozygosities (*H*_exp_). Linkage disequilibrium was tested among all pairs of loci with a permutation test (*n* = 1000). GENETIX was used to compute single and multilocus *F*_IS_ on the basis of the estimator of Weir and Cockerham (1984), and its significance was tested with 1000 permutations. The HP‐Rare software (Kalinowski, 2005) was used to estimate allelic richness [Ar(g)] and private allelic richness [Ap(g)] with a rarefaction analysis and 18 as minimum sample size. Differences in genetic diversity and allelic richness were tested between groups of populations, using the one‐sided probability test implemented in FSTAT 2.9.3.2 software (Goudet, 2001). The groups of populations were defined on the basis of geographical location, and of clustering analyses, and were Turkey, Algeria, France, and islands (Corsica and Menorca).

### 2.4 Demographic history

In order to test whether the analyzed population underwent recent population changes, we used two different approaches. First, the null hypothesis of mutation–drift equilibrium was tested using the software BOTTLENECK 1.2.02 (Piry, Luikart, & Cornuet, 1999). The tests were based on 1000 replicates under a two‐phase mutation model (TPM) with 95% of the stepwise mutation model (SMM) and variance among multiple steps equal to 12 (Cornuet & Luikart, 1996). Second, we used the MSVAR 1.3 software to evaluate the most probable demographic history on the basis of Markov Chain Monte Carlo (MCMC) simulations (Beaumont, 1999). First, we tested the sensitivity of the software to different starting points concerning ancestral and current effective sizes (respectively *N*_anc_ and *N*_curr_) on one population: We used either the same distributions for *N*_anc_ and *N*_curr_, or distributions indicating either reduction or expansion of populations. As this led to similar results indicating population decline (data not shown), we focused for the main analyses on an approach without a priori, with the same distributions for *N*_anc_ and *N*_curr_. Considering computation time, we analyzed separately a subset of two samples per area: KIA/DDC (pooled) and SPI for Algeria; MJS and RIS for France; SIV and AYV for Turkey; and MEN and REV for the islands group. The two Algerian samples KIA and DDC were pooled according to a nonsignificant differentiation comparison (see results). For continental France, the two retained samples corresponded to two depths and sites, and gave different results with BOTTLENECK. We also analyzed each region separately by grouping the corresponding population samples. In order to evaluate the impact of mutation model on the obtained results, we analyzed the results for the seven loci separately in the Algerian region. As genetic structure can impact the results of MSVAR analysis, an analysis at the deme level inside the French region was launched using two population samples in that region. We also pooled two individuals from the 12 population samples (total: 24 individuals) from the Marseille area, as an approximation to the method proposed by Chikhi, Sousa, Luisi, Goossens, and Beaumont (2010). This was not done in other areas because of a reduced number of independent samples. The parameters used for the MSVAR analyses are provided in Appendix S1 (Tables S2 and S3). We ran four independent chains with identical priors and starting values for each region. Each chain led to 20 000 lines of output. We tested the convergence of the MCMC chains with the Brooks, Gelman, and Rubin statistic (Brooks & Gelman, 1998; Gelman & Rubin, 1992). Values of the multivariate Gelman and Rubin's convergence diagnostic between 1.0 and 1.1 indicate reasonable convergence, whereas values >1.1 indicate poor convergence. In this regard, the last 10 000 output lines of each chain were retained to make a combined consensus chain of 40 000 data points for each region, which was assumed to summarize the posterior distribution of *N*_anc_ and *N*_curr_ (Storz & Beaumont, 2002). The output of MSVAR was analyzed by focusing on the detection and on the direction of demographic changes (expansion or contraction). We also compared the magnitude of changes between regions using both natural (*N*_curr_, *N*_anc_) and scaled parameters (θ_curr_ = 4*N*_curr_μ, θ_anc_ = 4*N*_anc_μ) over the four replicated data sets. All outputs were analyzed with the R CODA package (Plummer, Best, Cowles, & Vines, 2006).

### 2.5 Genetic structure

Pairwise *F*_ST_ were calculated with GENETIX according to Weir and Cockerham (1984). Their significance was tested with 1000 permutations. The excluding null allele (ENA) method in FreeNA (Chapuis & Estoup, 2007) was used to calculate pairwise *F*_ST_ to avoid potential bias induced by null alleles. As a complementary estimate of genetic differentiation, we computed the Jost's D statistic (Jost, 2008) with the SMOGD software (Crawford, 2010).

The pattern of isolation by distance (IBD) at the Mediterranean scale and within French region only (thanks to the number of samples in this region) was tested through the correlation between *F*_ST_/(1‐*F*_ST_) and the logarithm of geographical distances (shortest distance by sea) between populations (Rousset, 1997). The correlation was tested with a Mantel test (*n* = 10 000 permutations) in IBDWS 3.16 (Jensen, Bohonak, & Kelley, 2005).

An analysis of molecular variance (AMOVA) was performed with *F*_ST_ and *R*_ST_ estimators with ARLEQUIN v.3.5 (Excoffier & Lischer, 2010) and by using the main geographical areas as groups, that is, Turkey, Algeria, continental France, and Menorca and Corsica islands. For these last two islands, we conducted the AMOVA both by separating and by grouping them, as the STRUCTURE analysis grouped them (see results). One thousand permutations were used to test the significance of the different estimates of fixation indices of the AMOVA.

The relationships between populations were further investigated using principal coordinate analysis (PCoA) with GenAlEx 6.5 (Peakall & Smouse, 2006, 2012) using pairwise population matrix of Nei's unbiased genetic distance (Nei, 1972).

A clustering analysis was performed with the Bayesian method implemented in STRUCTURE v.2.2 (Falush, Stephens, & Pritchard, 2003, 2007; Pritchard, Stephens, & Donnelly, 2000) launched with admixture model, 500 000 iterations after a burn‐in period of 50 000, and 12 replicates for each configuration. A first round of analyses was launched with the whole data set to assess structure at the Mediterranean scale with *K* varying from 1 to 16. A second round of analyses was performed on each genetic group depicted by the initial round with the same parameter set of the first round, and *K* varying from 1 to 12 for France, and 1 to 5 in other cases. The outputs were analyzed through the STRUCTURE HARVERSTER website (Earl, 2012) to choose the value that captured the major structure in the data. The number of clusters was estimated based on the Delta (*K*) criterion (Evanno, Regnaut, & Goudet, 2005).

Results for each *K* value were merged with CLUMPP v.1.1 (Jakobsson & Rosenberg, 2007), and DISTRUCT v.1.1 (Rosenberg, 2004) was used to visualize these results.

To analyze genetic structure without relying on the model implemented in STRUCTURE, we performed a discriminant analysis of principal components (DAPC; Jombart, Devillard, & Balloux, 2010) implemented in the adegenet R package (Jombart, 2008). Data were analyzed in two rounds, one with all samples and a second round with French samples only. The number of clusters was determined based on the Bayesian information criterion (BIC).

In all cases, for multiple tests, significance levels were corrected using a 5% false discovery rate (FDR) (Benjamini & Hochberg, 1995).

## 3 Results

### 3.1 Genetic diversity

The total number of alleles per locus ranged from eight for Ever007 to 40 for Mic56 and a mean value of 18 alleles (Table 1). No evidence for null alleles, large allele dropout, or scoring errors due to stutters was found using MICRO‐CHECKER. No significant linkage disequilibrium among loci was generalized among populations (*p* > .05 after FDR correction). Observed and unbiased expected heterozygosities ranged from 0.31 for SIV to 0.62 for KIA, and from 0.34 for SIV to 0.71 for DDC, respectively (mean values: 0.48 and 0.56, respectively) (Table 1). Over all loci, significant heterozygote deficiencies were found in 15 samples of 19 (after FDR correction) with multilocus F_IS_ values ranging between 0 for AYV and 0.38 for 3PP (mean: 0.14; Table 1). Polymorphism data per locus and population are detailed in Appendix S2 and Table S2. Allelic richness Ar(18) ranged from 2.45 for SIV (Turkey) to 5.64 for DDC (Algeria; Table 1) and private allelic richness Ap(18) from 0.03 for RIS (Marseille) to 1.13 for KIA (Algeria). Turkish populations presented significantly lower expected heterozygosities and allelic richness compared to other groups of samples, with *H*_exp_ = 0.44 for AYV and 0.34 for SIV, and Ar = 2.6 for the Turkish group, compared to 4.2 for Marseille and the group of Menorca and Corsica islands, and 5.2 for Algeria (Table 2). The two groups of Marseille and of Menorca and Corsica islands showed significantly lower genetic diversity than Algeria, which was the most diverse area for all criteria.

**Table 2:** Differences in genetic diversity and allelic richness between groups of populations, using one‐sided probability test (G1 indicates the diversity of the first region and G2 of the second region)

Comparison	Observed values			One‐sided *p*‐values (G1 > G2)		
	Allelic richness	*H*_obs_	*H*_exp_	Allelic richness	*H*_obs_	*H*_exp_
Algeria	5.2	0.57	0.68	0.014	0.01	0.008
France	4.3	0.47	0.56			
Algeria	5.2	0.57	0.68	0.001	0.001	0.001
Turkey	2.6	0.38	0.39			
Algeria	5.2	0.57	0.68	0.085	0.199	0.094
Islands	4.2	0.51	0.57			
France	4.3	0.47	0.56	0.004	0.043	0.004
Turkey	2.6	0.38	0.39			
France	4.3	0.47	0.56	0.455	0.775	0.584
Islands	4.2	0.51	0.57			
Islands	4.2	0.51	0.57	0.02	0.027	0.015
Turkey	2.6	0.38	0.39			

### 3.2 Demographic fluctuations

**Figure 3:**
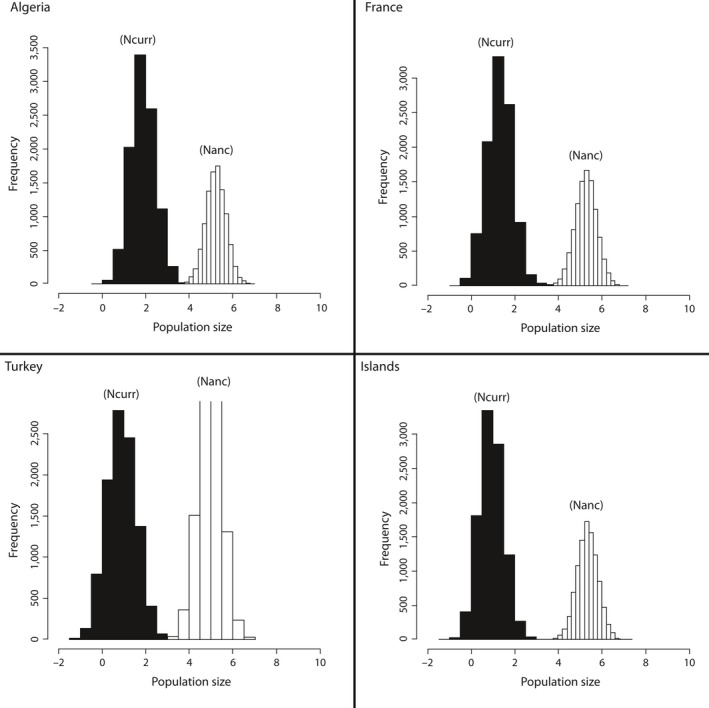
Marginal posterior density of current and ancestral population size in four regions of the Mediterranean. Densities are expressed in a log_10_ scale

The analysis of departure from mutation–drift equilibrium using BOTTLENECK indicated no significant heterozygosity excess, expected following a bottleneck, on the basis of one‐tailed Wilcoxon test (Table 3). On the other hand, significant heterozygosite deficiency, expected after population expansion, was detected in 10 samples over 19, and nine tests remained significant after FDR correction. These signals of expansion were observed in five over twelve northern populations (Marseille) and in Algeria and Balearic Islands. Contrastingly, MSVAR results indicated a strong historical decline for all the analyzed samples whether separately (Appendix S3, Figure S1, Table S1) or pooled per region (Figure 3; Appendix S3, Table S2). At the region level, the inferred *N*_anc_/*N*_curr_ reached 10^4^ in Turkey and around 2 × 10^3^ in Algeria (Appendix S3, Table S2). When considering the scaled parameters, the current value of θ = 4Neμ was again highest in Algeria, intermediate in France and islands, and lowest in Turkey. The θ estimate was ten times higher in Algeria than in Turkey (0.21 vs 0.02, respectively; Table 4; Appendix S3, Figure S2). Current effective sizes were also lower in Turkish samples than in other samples at deme level (mean values: 0.78 for AYV, and 0.67 for SIV; Appendix S3, Table S1). In the French region with a pool of 24 individuals, two for each site, results also indicated a population decline, but at a lower intensity than with the regional analysis with two demes (*N*_anc_/*N*_curr_ around 300 and 9 × 10^3^ for the pool and the regional analysis, respectively; Appendix S3, Table S3, Figure S3). The inference of demographic decline was coherent along all our loci in the Algerian area, all indicating signatures of population declines (Appendix S3, Figure S4, Table S4).

**Table 3:** Tests of mutation–drift equilibrium in *E. cavolini* with BOTTLENECK

Pop	Probability		Region
	*H*_def_	*H*_exc_	
KIA	**0.00**	1.00	Algeria
SPI	**0.02**	0.98	
DDC	**0.01**	0.99	
MEN	**0.02**	0.99	Balearic Islands
REV	0.23	0.81	Corsica
CAV	**0.00**	1.00	France—Provence
MEJ	**0.02**	0.99	
MJS	**0.01**	1.00	
POU	0.05	0.96	
RID	0.19	0.85	
RIS	0.41	0.66	
SAM	0.05	0.96	
SOM	**0.02**	0.99	
VED	0.05	0.96	
VES	0.04	0.97	
3PP	0.05	0.96	
SAR	**0.01**	0.00	
AYV	0.36	0.72	Turkey
SIV	0.50	0.59	

*p* values for the one‐tailed Wilcoxon test for heterozygosity excess (*H*_exc_) and deficiency (*H*_def_). Significant values in bold.

**Table 4:** Scaled parameter estimates (θ_curr_ = 4*N*_curr_μ, θ_anc_ = 4*N*_anc_μ, *t* = *T*(2*N*_curr_)) in four regions of the Mediterranean

	Algeria			France			Islands			Turkey		
	θ_curr_	θ_anc_	*t*	θ_curr_	θ_anc_	*t*	θ_curr_	θ_anc_	*t*	θ_curr_	θ_anc_	*t*
Mean	0.21	398.11	5.25	0.04	380.19	10.96	0.04	389.05	12.59	0.02	147.91	20.89
Media	0.22	407.38	5.25	0.04	398.11	11.22	0.04	407.38	12.88	0.03	154.88	20.89
*SD*	2.24	1.35	1.41	2.69	1.58	1.62	2.51	1.48	1.35	3.63	2.14	1.58

### 3.3 Genetic structure

Pairwise *F*_ST_ values ranged from 0 (KIA vs DDC; RID vs RIS; POU vs RID; POU vs RIS) to 0.36 (SAR in France vs SIV in Turkey; Appendix S2, Table S3) with an overall *F*_ST_ = 0.13. All populations were significantly differentiated except KIA and DDC (Algeria) that are separated by a distance of 721 m, and in Marseille for samples from the same site but different depths (MEJ vs MJS, VES vs VED, and RID vs RIS). A sample from Marseille (POU) with 25% missing data showed also five nonsignificant differentiation tests (POU vs VES/VED, POU vs RID/RIS, and POU vs 3PP) after FDR correction. Pairwise F_ST_ values corrected for null alleles showed similar values of differentiation between samples (Appendix S2, Table S4). The smallest geographical distance for which significant genetic differentiation was observed was 763 m, with *F*_ST_ = 0.02 for KIA vs SPI.

The AMOVA indicated significant differences between geographical groups of samples, both by separating the two islands (Table 5) and by grouping them (Appendix S2, Table S5). For the analysis separating the two islands, differences among groups were significant (*F*_CT_ = 0.19 and *F*_CT_ = 0.04 with *F*_ST_‐like and *R*_ST_‐like analyses, respectively; Table 5). The differences between populations within groups appeared significant with *F*_ST_ but not significant with *R*_ST_ (*F*_SC_ = 0.03 and −0.01, respectively). A significant positive correlation was evident between genetic distances and the logarithm of the geographical distances, indicating a pattern of IBD at the Mediterranean scale (*R*² = .567, *p* < .0001; Appendix S2, Figure S1) and within the French region (*R*² = .169, *p* = .009; Appendix S2, Figure S2). At the global scale, the IBD pattern seemed to be separated in two parts with a lower slope at short distance compared to a much higher slope at higher distances.

**Table 5:** Results of AMOVA. The groups of populations were defined on the basis of geographical location and of clustering analyses and were Turkey, Algeria, France, and islands (Corsica and Menorca)

Source of variation	*R*_ST_‐like analysis		*F*_ST_‐like analysis	
	Percentage of variation	Fixation index	Percentage of variation	Fixation index
Among groups	6.06%	*F*_CT_ = 0.04 (*p* = .03)	16.22%	*F*_CT_ = 0.19 (*p* = 0)
Among populations within groups	0%	*F*_SC_ = −0.01 (*p* = .99)	2.96%	*F*_SC_ = 0.03 (*p* = 0)
Within populations	95.47%	*F*_ST_ = 0.06 (*p* = 0)	80.82%	*F*_ST_ = 0.16 (*p* = 0)

The significance of the different parameters was tested with permutations (*n* = 1000 of each analysis).

**Figure 4:**
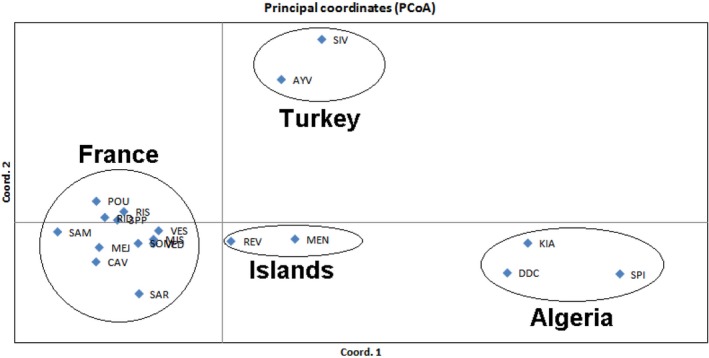
Plot of the first two axes from the principal coordinate analysis based on Nei's unbiased genetic distance. Percentage of variation explained by axis 1: 51.1. and by axis 2: 22

The principal coordinate analysis (PcoA) confirmed the distinctiveness of the different geographical areas: Axis 1 separated Algeria from other samples, while axis 2 separated Turkey from other ones (Figure 4). Island samples (Balearic and Corsica) appeared closer to French samples than to other areas but well separated from them.

### 3.4 Clustering analysis

**Figure 5:**
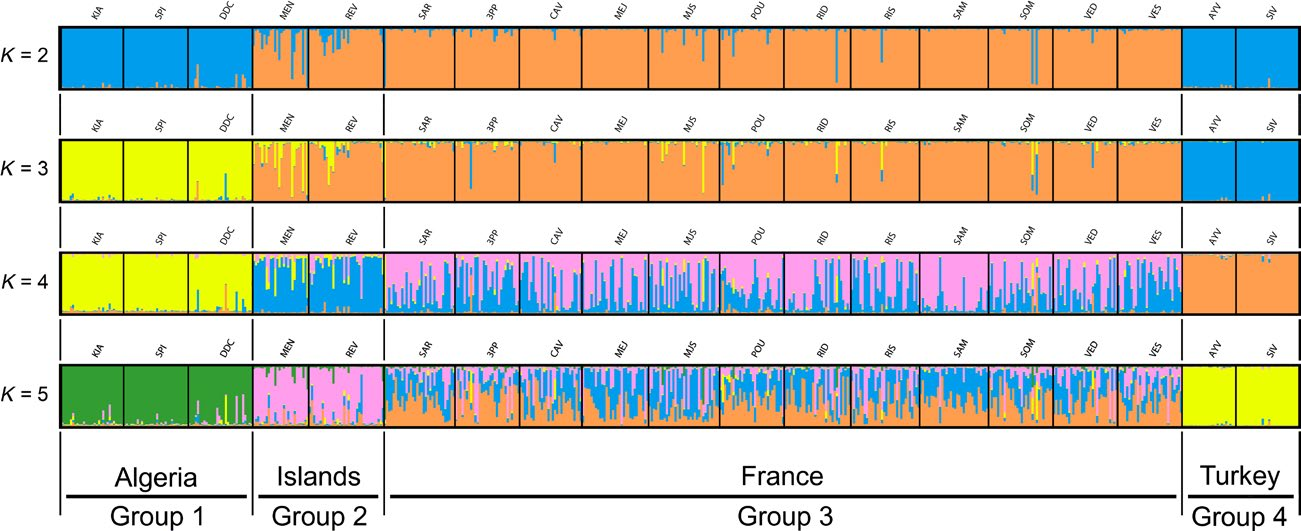
Bar plot from the first round of analysis with STRUCTURE, revealing population structure of *Eunicella cavolini* at the Mediterranean scale with retained values of *K* = 2, 3, 4, and 5. One bar corresponds to one individual, and the colors correspond to the different clusters. The proportion of color for each individual corresponds to its membership probabilities for the corresponding clusters

The first round of STRUCTURE with *K* = 2 separated samples from Algeria and Turkey in cluster 1 and samples from France, Menorca, and Corsica in cluster 2 (Figure 5). For *K* = 3, samples from France, Menorca, and Corsica were assigned to cluster 1, while samples from Algeria and Turkey were separated in two different clusters (2 and 3, respectively). At *K* = 3, four replicates over twelve grouped Algerian and Turkish samples and were not retained here. For *K* = 4, samples from islands Menorca and Corsica were assigned to a new group, while other samples were clustered as above but with France partly admixed with the islands cluster (Figure 5). The Delta(*K*) criterion indicated *K* = 5 as the best clustering solution (Appendix S2, Figure S3). With *K* = 5, Algerian samples were in cluster 1, Menorca and Corsica in cluster 2, Turkey in cluster 3 and all French samples subdivided between clusters 4 and 5 but with high admixture between these two putative clusters.

For the second STRUCTURE analysis on French samples, *K* = 3 was the best solution followed by *K* = 6 (Appendix S2, Figure S4), but there was no clear genetic structuring (Appendix S2, Figure S5). A STRUCTURE analysis on Turkish samples alone indicated a clear separation of both populations at *K* = 2 (Appendix S2, Figure S6). The STRUCTURE analysis on Menorca and Corsica samples indicated a distinction between these two islands but with quite high levels of admixture (Appendix S2, Figures S7 and S8).

**Figure 6:**
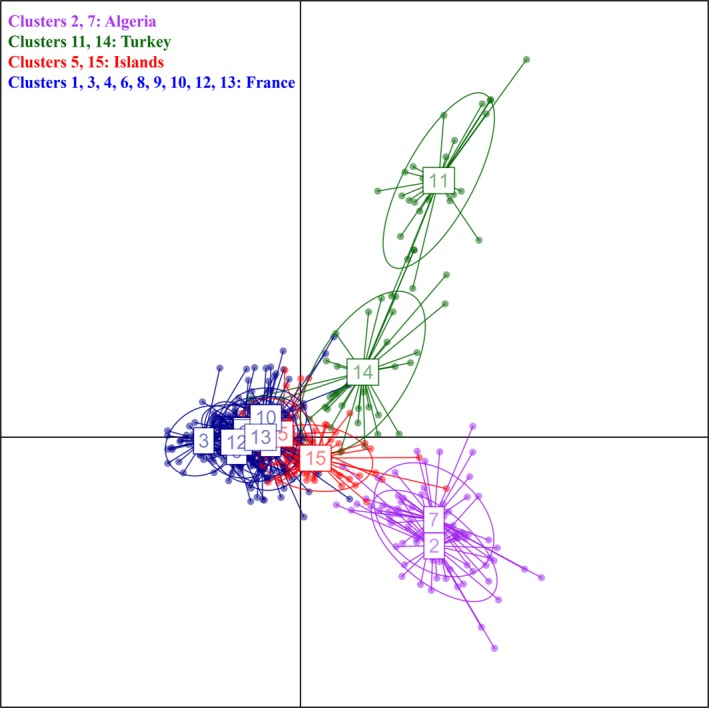
Results of the DAPC analysis of *Eunicella cavolini* with *K* = 15 genetic clusters. Main regions of the Mediterranean are separated by different colors. Green corresponds to Turkish samples, purple to Algerian samples, blue to French samples, and red to the samples from Corsica and Menorca

For the DAPC analysis, the Bayesian information criterion (BIC) was minimal between *K* = 15 and 21 but without a single clear informative value (Appendix S2, Figure S9). We present here the results obtained with *K* = 15 clusters, and other analyses around this value gave similar results. Higher *K* values did not bring more information on the general structure at the Mediterranean scale. The analysis confirmed the main groupings evidenced with STRUCTURE but with the additional separation between the two Turkish samples from Marmara Sea and Aegean Sea in clusters 11 and 14, respectively (Figure 6). Samples from Algeria were assigned to clusters 2 and 7. The two samples of Menorca and Corsica were mainly grouped in clusters 5 and 15, respectively, while French samples were mainly assigned to the remaining clusters (Table 6). High percentages (>0.70) of reassignment to the original clusters were observed apart from clusters 1, 4, and 10 corresponding to samples from France (Appendix S2, Table S6). A second DAPC analysis on French samples only did not indicate any clear additional substructuring (Appendix S2, Figures S10 and S11).

**Table 6:** Results of population assignments in 15 clusters using DAPC analysis

		Clusters															*N*
		1	2	3	4	5	6	7	8	9	10	11	12	13	14	15	
Algeria	KIA	1	10	0	0	0	0	15	0	0	0	0	0	0	1	3	30
	SPI	0	13	0	0	1	0	15	0	0	0	0	0	0	0	1	30
	DDC	0	9	0	0	1	0	16	0	0	0	0	0	0	2	1	29
Islands	MEN	1	1	0	0	6	0	0	0	0	0	0	0	2	0	16	26
	REV	4	0	0	2	8	4	2	2	0	1	0	0	1	0	11	35
France	CAV	4	0	6	4	4	5	0	0	0	0	0	5	1	0	0	29
	MEJ	7	0	0	4	2	2	0	3	2	2	0	6	2	0	1	31
	MJS	6	1	0	4	4	3	0	1	0	4	0	4	6	0	0	33
	POU	7	0	2	4	7	0	0	3	1	4	0	0	2	0	0	30
	RID	3	0	5	5	0	2	0	1	7	1	0	0	5	0	2	31
	RIS	2	0	1	5	6	1	0	1	6	3	0	3	1	1	2	32
	SAM	0	0	2	0	2	8	0	3	11	1	0	2	2	0	1	32
	SOM	7	0	5	1	4	4	0	4	0	0	0	0	0	3	2	30
	VED	7	0	3	6	2	0	0	2	0	5	0	0	1	1	3	30
	VES	5	0	4	4	4	1	0	2	0	2	0	0	3	1	4	30
	3PP	2	0	1	3	2	2	0	9	1	9	0	0	0	0	1	30
	SAR	9	1	2	5	2	6	0	0	1	2	0	4	0	0	1	33
Turkey	AYV	0	0	0	0	1	0	0	0	0	1	0	0	0	23	0	25
	SIV	0	0	0	0	0	0	0	0	0	0	25	0	0	4	0	29

*N,* number of individuals.

## 4 Discussion

We have demonstrated (1) strong genetic structure between samples from different regions in the Mediterranean, (2) we did not observe any significant differentiation between depths for a given site in France, and (3) we have shown significant differences in the levels of genetic diversity between regions with the highest values in southwestern Mediterranean (Algeria) and the lowest in the eastern part (Turkey), which could be correlated to different evolutionary histories or levels of effective sizes.

### 4.1 Genetic structure of *E. cavolini* and comparison with other Mediterranean octocorals

We identified four main clusters corresponding to geographical subdivisions: northwestern Mediterranean, Balearic and Corsica islands, and Algeria and Turkish samples. These differences between regions were statistically significant. These results can be discussed in the more general context of the biogeography of the Mediterranean Sea. *F*_ST_ values indicated that the highest differentiation was observed between eastern (Turkish) and western populations and differentiation was higher when comparing western samples with Marmara Sea (mean *F*_ST_ = 0.3) than with Aegean Sea (mean *F*_ST_ = 0.22). Such deep genetic break between eastern and western Mediterranean populations has been reported in various species such as fish, molluscs, or the seagrass *Posidonia oceanica* (Arnaud‐Haond et al., 2007; Bahri‐Sfar, Lemaire, Hassine, & Bonhomme, 2000; Nikula & Väinölä, 2003). An east–west divergence has been observed for the octocoral *P. clavata* but with only one sample in the eastern basin (Mokhtar‐Jamaï et al., 2011). The Siculo‐Tunisian strait is indeed considered as an important genetic boundary for various marine species (Borsa et al., 1997). Concerning *E. cavolini,* the strong differentiation between eastern and western Mediterranean samples could be explained by several potential oceanographic barriers, including the Siculo‐Tunisian strait, but their exact location remains to be studied (Berline, Rammou, Doglioli, Molcard, & Petrenko, 2014). Additionally, the gaps in the distribution range of *E. cavolini* between Turkey and Algeria could contribute to this differentiation (Sini, Kipson, Linares, Garrabou, & Koutsoubas, 2014). Isolation by distance could lead to the identification of well‐separated clusters as well, if distant populations are analyzed without geographical intermediates (Aurelle & Ledoux, 2013).

The North/South differentiation evidenced here has not often been observed in Mediterranean phylogeographic studies but has been tested in three other Mediterranean octocorals. For *P. clavata,* the southern samples appeared well differentiated from northern ones. But these southern samples were also situated on the western side of the Almeria–Oran front which might contribute to this differentiation (Mokhtar‐Jamaï et al., 2011). For *C. rubrum,* northern samples appeared well separated from southern ones (Algeria and Morocco, including samples from the Atlantic side of the Almeria–Oran front; Aurelle et al., 2011). Conversely, the Algerian populations of *E. singularis* were partly related to northern samples of the French coasts (near Spain; Cataneo, 2011). The discrepancy between *E. singularis* and *E. cavolini* might be related to a lower number of loci in the former compared to our study (five compared to seven), or to intrinsic different patterns of genetic structure. Here again, the distribution range of *E. cavolini* could partly explain these differences between *E. singularis* and *E. cavolini*: There is a notable absence of *E. cavolini* along Mediterranean Spanish coasts. This could have promoted a North–South coastline connectivity for *E. singularis* which is more frequent there (Sini et al., 2014). The genetic structure of *E. cavolini* should be studied along the Italian coasts to test the possibility of connectivity by this way. This North–South differentiation could also correspond to a particular isolation of Algerian populations because of mesoscale eddy systems as proposed for the dusky grouper *Epinephelus marginatus* (Schunter et al., 2011). Nevertheless, this hypothesis alone would not explain the contrasted results obtained for different *Eunicella* species. In all cases, our results and most previous studies point to the genetic distinctiveness of southern octocoral communities.

The samples from Balearic (Menorca) and Corsica islands were identified as a separate cluster without substructure (Appendix S2, Figure S8). The F_ST_ between these samples was significant but lower than those observed for some comparisons in the Marseille area for example. The grouping of Menorca samples with Corsica was not consistent with the Mediterranean current clusters defined by Berline et al. (2014). The geographical distance and lack of shallow benthic habitats between them are additional factors which could promote divergence. Accordingly, clustering analyses on other octocorals (*C. rubrum* and *P. clavata*) displayed a genetic distinction between Balearic Islands and Corsica (Ledoux et al., 2010; Mokhtar‐Jamaï et al., 2011). The results obtained for *E. cavolini* could point to a recent divergence between island populations from a common ancestral population.

The Turkish samples from Marmara Sea and Aegean Sea appeared strongly differentiated according to *F*_ST_ value (0.24) and to DAPC. This was not visible with the global STRUCTURE analysis probably because of the reduced number of Turkish samples analyzed. Indeed unbalanced sampling affects such clustering method (Aurelle & Ledoux, 2013). However, the STRUCTURE analysis on Turkish samples alone clearly identified a genetic break here. A genetic differentiation between Black Sea and the Mediterranean Sea has been demonstrated for example for the mussel *Mytilus galloprovincialis* (Ladoukakis, Saavedra, Magoulas, & Zouros, 2002), or the anchovy *Engraulis encrasicolus* (Magoulas, Castilho, Caetano, Marcato, & Patarnello, 2006). Nevertheless, in most cases, the precise location of the genetic break could not be determined, especially its position relative to the Marmara Sea which connects Black Sea and Aegean Sea. Here, we demonstrate an important genetic differentiation between the Marmara Sea and the Aegean Sea for *E. cavolini*. The Marmara Sea presents a particular circulation pattern with shallow, low‐salinity, waters coming from the Black Sea and deeper salty waters coming from the Mediterranean (Beşiktepe et al., 1994). This, along with the strait systems delimiting the Marmara Sea, provides a strong isolating factor for octocorals, which are restrained to deeper locations.

In all cases, considering the important differentiation observed between some of these clusters, especially the eastern–western differentiation, genetic incompatibilities may contribute to the observed differentiation as well (Bierne, Welch, Loire, Bonhomme, & David, 2011). Genome scan approaches would be useful here to go further on this topic.

### 4.2 Genetic structure at regional and local scales

In the Marseille area, the maximum pairwise F_ST_ reached 0.07 for populations separated by 15 km. Such local genetic structure has been demonstrated for other octocorals in this area, with maximum *F*_ST_ reaching 0.2 for *C. rubrum* (Ledoux et al., 2010) and 0.1 for *P. clava*ta (Mokhtar‐Jamaï et al., 2011). This has been linked to reduced dispersal abilities of the larval stage in Mediterranean octocorals (Martínez‐Quintana et al., 2015). In *E. verrucosa,* the lecithotrophic larvae are supposed to have a short, but unknown life span (Sartoretto & Francour, 2011). In *E. singularis*, experimental results indicated that in the presence of favorable substrates, settlement could take place in less than 30 hr (Weinberg & Weinberg, 1979). If similar larval traits are present in *E. cavolini*, this could explain, along with important genetic drift, our observation of a strong local genetic structure.

No significant differentiation was evidenced between samples from different depths within the same sites near Marseille as observed in a preliminary study (Pivotto et al., 2015). This suggests the occurrence of regular gene flow or low genetic drift that leads to a genetic homogeneity between depths. This was also observed for *E. singularis* (Cataneo, 2011), but it contrasts with previous findings of genetic structure between depths for *C. rubrum* (Costantini et al., 2011; Ledoux et al., 2010) and *P. clavata* (Mokhtar‐Jamaï et al., 2011). Such differences between species could be linked to the buoyancy or the vertical movements of the larvae of these species. The precise timing of larval release, relative to the onset of thermocline, could explain these results and would require a precise study of phenology according to water stratification. The observation of a lack of genetic differentiation between depths despite clear thermotolerance differences questions the possibility of local adaptation in *E. cavolini* (Pivotto et al., 2015). In the Carribean octocoral *Eunicea flexuosa*, adaptation to different depths coincided with distinct genetic lineages (Prada & Hellberg, 2013). For *E. cavolini,* an intron locus seems to indicate significant differences according to depth and could be linked to a selected polymorphic locus (Aurelle et al. submitted). It will thus be necessary to study more loci to test for possible genetics–environment associations.

### 4.3 Contrasting results between BOTTLENECK and MSVAR approaches

Concerning demographic history, BOTTLENECK tests and estimates of past versus current effective sizes gave contrasting results. Whereas BOTTLENECK indicated either no demographic fluctuation or population expansion, the MSVAR approach suggested a generalized population decline with different strengths. Such discrepancies between these two methods have been demonstrated by Girod et al. (2011): In a simulation of population decline, these authors observed that MSVAR could indeed detect the correct demographic change, but in some simulation cases with the oldest changes, BOTTLENECK suggested a population expansion. We provide here additional empirical evidence of contrasting results between tests and model‐based studies of demographic history. The MSVAR analysis can lead to false inferences of population decline in cases of strong departures from a stepwise mutation model (SMM; Girod et al., 2011; Faurby & Pertoldi, 2012) or in case of underlying genetic structure (Chikhi et al., 2010). Concerning departures from SMM, the inference of demographic decline was coherent along all our loci which present different levels of variability and distributions of allele sizes. Regarding genetic structure, the analysis at the deme level inside regions gave similar results to pooled samples, but the analysis of a single deme can lead to spurious inference of decline as well (Chikhi et al., 2010). A decline, though less strong, was also inferred for a pool of individuals scattered along different demes as suggested by Chikhi et al. (2010). These observations suggest that *E. cavolini* populations were indeed impacted by a demographic decline, but the estimates of the magnitude of this decline may be biased by population structure. As suggested by Faurby and Pertoldi (2012), we focus the following interpretation of MSVAR results on the inferred relative levels of current effective size. At the deme level, the inferred current effective sizes were much lower in Turkey samples than in other Mediterranean areas.

### 4.4 Genetic diversity and inferences on evolutionary history

*E. cavolini* is a high diversity species among metazoans (Romiguier et al., 2014). We evidenced here that this diversity is highly heterogeneous among regions as a potential result of differences in effective sizes, a result which remained robust when considering estimates of scaled effective size. Turkish samples displayed the lowest levels of genetic diversity, whereas Algerian samples displayed the highest diversity (a twofold difference in allelic richness and a 42% reduction in expected heterozygosity in Turkey compared to Algeria).The lower reduction in heterozygosity compared to allelic richness is expected as a loss of rare alleles has a higher impact on the latter. Differences in the levels of genetic diversity between populations can be the consequence of different evolutionary histories (eg, bottleneck) and on differences in effective size for a same history. The lowest levels of genetic diversity in Turkey can be discussed according to the peculiar history of the Marmara Sea. Several scenarios of connection between Black Sea and Mediterranean Sea after the last glacial maximum have been proposed. Ryan et al. (1997) suggested that during the last glacial maximum, the Black Sea became a giant freshwater lake and that the Mediterranean Sea refilled it. A second hypothesis suggested that it was instead the Black Sea that first breached the Bosphorus and overflowed into the Marmara Sea (Aksu et al., 2002). In both cases, they imply recent colonization events for the Marmara Sea and possibly for the neighboring part of the Aegean Sea. A reduced diversity of Turkish populations compared to Western Mediterranean populations has also been observed in the red gorgonian *P. clavata*, but with only one Turkish population considered (Mokhtar‐Jamaï et al., 2011). The population density of *E. cavolini* in the neighboring northern Aegean is also lower than in western Mediterranean, and recruitment frequency seems reduced in eastern populations as well (Sini, Kipson, Linares, Koutsoubas, & Garrabou, 2015). This agrees well with our inferences of lower current effective size and lower diversity in this area. The high level of private allelic richness (second after Algeria) in this area also points to an historical isolation of these populations (Waples, 2010) which could have strengthened the regional loss of diversity.

The highest diversity was observed in Algeria, but no demographic studies have been carried out for southern populations for comparison with northern ones. The highest private genetic richness observed here also underlines the isolation of this southern cluster. This could indicate that this area corresponded to a glacial refugia (Maggs et al., 2008) or, at least, to an area where the environmental conditions would have allowed a better demographic stability of these populations than in other regions. Quaternary climatic fluctuations led to variations in sea temperature and sea level. Winter surface temperature was estimated to be around 7°C in winter in the Gulf of Lion (compared to 13°C for present day) and the sea level was 120 m lower than present around French coasts (Hayes et al., 2005; Lambeck & Bard, 2000). These variations could have had different demographic impacts according to location, as the cooling was lower along Algerian coasts (Hayes et al., 2005). If southern areas were refugia for *E. cavolini*, the recolonization of northern Mediterranean could have led to a “southern richness to northern poverty” (Hewitt, 2000). For *E. cavolini*, the marked differentiation between northern and southern populations could point to an alternative scenario: There could be a northern refugia more affected by climatic fluctuations than southern one. A higher diversity in southern compared to northern populations has not been observed for other octocoral populations, apart for *E. singularis* (Aurelle et al., 2011; Cataneo, 2011; Mokhtar‐Jamaï et al., 2011). This could suggest different responses of octocorals to climate fluctuations with a higher sensitivity of *E. cavolini* to past climatic variations.

## 5 Conclusion

The yellow gorgonian *E. cavolini* presents distinct genetic units depending on geographical locations with contrasted levels of genetic diversity. Therefore, protection of genetically rich populations (eg, in Algeria) or with an important private diversity (eg, in Turkey) should be a priority. This is particularly important when considering the current pressures on this species. In Turkey, some relatively dense *E. cavolini* populations, restricted to few areas in the Marmara Sea, are under various threats such as fisheries (Topçu & Öztürk, 2015). Their genetic particularity could make these populations more vulnerable against such threats that tend to lower their abundance. The impact of local environmental conditions on such species should be considered for protection. Genomic studies of this species could open the way to a better understanding of its evolution and adaptation in a heterogeneous and fluctuating environment. It would also be useful for a better understanding of the evolutionary history of this species.

## Data accessibility

All genotypes are deposited on Dryad. doi:10.5061/dryad.8vj25.
