# Supplementary material for: Contrasted levels of genetic diversity in a benthic Mediterranean octocoral: Consequences of different demographic histories?
Source: Ecol Evol. 2016 Oct 28;6(24):8665–78. doi: 10.1002/ece3.2490 (PMC5192949; doi:10.1002/ece3.2490)
Supplement: Supplementary file 1 [file ECE3-6-8665-s001.docx]

**Appendix S1.**

**Development of a new microsatellite marker**

The Locus Mic56 was identified through the sequencing on an intron from the putative Glutamyl-prolyl-tRNA-synthetase gene. This intron was first sequenced with the primers i56 developed by Chenuil *et al.* (2010).

**Cross-amplification from other *Eunicella* species**

We tested other loci developed for the study of *E. singularis* and *E.* verrucosa. Loci C21, C30, C40, S14 have been developed for the study of *E. singularis* (Molecular Ecology Resources Primer Development Consortium *et al.* 2010). Locus S14 has been developed from *E. singularis* genome, whereas loci C21, C30 and C40 have been developed from *E. cavolini* but have not been tested in this species. We also tested the latter four loci on *E.verrucosa* by sampling 30 individuals in the Algerian region (Hauffman, 36°57'N 7°47'E), and performing cross amplification reliably (the number of alleles per loci ranged between 4 and 6, observed and expected heterozygosities ranged from 0.14 and 0.48, and between 0.44 and 0.79 respectively).

Loci Ever007 and Ever009 have been developed from genomic sequences of *E. verrucosa* by Holland *et al.* (2013) These authors only tested these loci on 10 and 12 individuals of *E. cavolini* for Ever 007 and Ever 009 respectively.

**Polymorphism analyses**

Among the 11 new loci isolated from *Eunicella* spp. transcriptomes, three amplified reliably and were selected for subsequent analysis (Table S1). Loci developed from other species were directly analyzed on an ABI 3130 Genetic Analyser. GeneMapper v.4.0 software was used to score alleles. The multiplex PCR were performed in 10 µL final volume with primers at 2 µM using the Qiagen Type-it microsatellite PCR kit. The PCR program was: 3 min at 94°C followed by 30 cycles with 1 min at 94°C, 1 min at annealing temperature and 1 min at 72°C; a final extension of 5 min at 72°C was added. Table S1 indicates the annealing temperature for all loci.

**References:**

Chenuil A, Hoareau TB, Egea E, Penant G, Rocher C, Aurelle D *et al* (2010). An efficient method to find potentially universal population genetic markers, applied to metazoans. *BMC Evol Biol* **10**(1)**:** 276.

Holland LP, Dawson DA, Horsburgh GJ, Krupa AP, Stevens JR (2013). Isolation and characterization of fourteen microsatellite loci from the endangered octocoral Eunicella verrucosa (Pallas 1766). *Conservation Genetics Resources* **5**(3)**:** 825-829.

Molecular Ecology Resources Primer Development Consortium, Abdoullaye D, Acevedo I, Adebayo A, Behrmann-Godel J, Benjamin RC, Bock DG *et al* (2010). Permanent genetic resources added to molecular ecology resources database 1 August 2009–30 September 2009. *Mol Ecol Resour*, **10**, 232–236.

**Table S1 :** Microsatellite characteristics: primer sequences (F: forward, R: reverse), repeat motif (inter: interrupted), fluorochrome used (for the retained loci), observed allele range. Annealing temperature for PCR. Reference: origin of the loci (MER 2010 refers to Molecular Ecology Resources Primer Development Consortium *et al.* 2010).

| **Locus** | **Primer sequence (5'-3')** | **Repeat motif** | **Fluo-Label** | **Annealing T°** | **Obs. allele size range (bp)** | **Reference** | **Isolated from** | **GenBank accession number** |
| --- | --- | --- | --- | --- | --- | --- | --- | --- |
| C21 | F:TGGGATGTCAAGTGGTTTTCAAG R:CCGGTTTCAGGTTCATGCC | (AAT)_10_ | 6FAM | 55° | 181-199 | MER 2010 | *E. cavolini* | - |
| C30 | F:TCAGCCCGAGTTGTTGAGGC R:ACGTAGCACCATAGTACCG | (ATGT)_5_ | NED | 62° | 170-202 | MER 2010 | *E. cavolini* | - |
| C40 | F:AGATGAGAAAGGACGGTCGG R:CACCAGCAACAACAACTGC | (GTT/GCT)_24_ inter | NED | 60° | 265-296 | MER 2010 | *E. cavolini* | - |
| S14 | F:TTGAAGGTGTGAACAACTACGG R:CTGGCTGGCTGTTGCTG | (AAC)_17_inter | PET | 64° | 146-256 | Cataneo *et al.* 2011 | *E. singularis* | - |
| MIC56 | F : TCTCATCAGATATTYTGATATRCA  R : AACAAGGTTAAAACACGGCATGCC | (CTT)_148_ | VIC | 56° | 220-532 | This study | *E. cavolini* | KP780074 |
| EVER007 | F:GGTAACAAACTTAGCACAGC R:GCTAATAATGAGCCAATCACCC | (GA)_10_ | 6FAM | Multiplex 57° | 225-240 | Holland *et al.* 2013 | *E. verrucosa* | - |
| EVER009 | F:ATACAAGTTCTGGTGGCATGG R:CCCTCCTGTAAATCAGCATATTG | (AATC)_8_ | 6FAM | Multiplex 57° | 93-120 | Holland *et al.* 2013 | *E. verrucosa* | - |

**Table S2 Settings for msvar:** Consensus chains for msvar were run modelling exponential decline, generation time five years and the following hyperpriors on parameters:

| Ploidy number | 2 |
| --- | --- |
| Generation time | 5 |
| Starting values for current size for all loci | 10^3^ (for all loci) |
| Starting values for ancestral size for all loci | 10^3^ (for all loci) |
| Starting values for time since decline/expansion for all loci | 4x10^3^ (for all loci) |
| Indicators (0,1) whether to update values of these parameters. | 0 |
| Starting values for prior mean / variance for current size | 3 / 1 |
| Starting values for prior mean / variance for ancestral size | 3 / 1 |
| Starting values for prior mean / variance for mutation rate | -3.3 / 1 |
| Number of lines of output | 20000 |
| Number of iterations between lines of output | 100000 |

**Table S3 :** Hyperprior settings in the consensus chains (notation following Storz et al., 2002, modified). Hyperprior parameters: α = mean of mean; σ = std. dev. of mean; β = mean of std. dev.; τ = std. dev. of std. dev.

|  | *α* | *σ* | *β* | *τ* |
| --- | --- | --- | --- | --- |
| *log N*_curr_ | 3 | 1.5 | 0 | 0.5 |
| *log N*_anc_ | 3 | 1.5 | 0 | 0.5 |
| *log* μ | -3.3 | 0.5 | 0 | 1 |
| *log* *T* | 3.6 | 2 | 0 | 0.5 |
