# Supplementary material for: Contrasted levels of genetic diversity in a benthic Mediterranean octocoral: Consequences of different demographic histories?
Source: Ecol Evol. 2016 Oct 28;6(24):8665–78. doi: 10.1002/ece3.2490 (PMC5192949; doi:10.1002/ece3.2490)
Supplement: Supplementary file 2 [file ECE3-6-8665-s002.docx]

**Appendix S2.**

**Table S1 :** Individuals presenting duplicated multilocus genotypes MLGs (eliminated from initial data set).

| **Individual** | **Region** |
| --- | --- |
| ECDDC25 | Algeria |
| ECSAM32 | France |
| ECSOM15 |  |
| ECSIV21 | Turkey |
| ECAYV12 |  |
| ECAYV21 |  |
| ECAYV22 |  |
| ECAYV26 |  |
| ECAYV30 |  |

**Table S2 :** Polymorphism data per locus and per population

|  |  | Populations | | | | | | | | | | | | | | | | | | |
| --- | --- | --- | --- | --- | --- | --- | --- | --- | --- | --- | --- | --- | --- | --- | --- | --- | --- | --- | --- | --- |
|  |  | 1 | 2 | 3 | 4 | 5 | 6 | 7 | 8 | 9 | 10 | 11 | 12 | 13 | 14 | 15 | 16 | 17 | 18 | 19 |
|  |  | KIA | SPI | DDC | MEN | 3PP | CAV | MEJ | MJS | POU | REV | RID | RIS | SAM | SAR | SOM | VED | VES | AYV | SIV |
| C21 | N | 3 | 4 | 3 | 3 | 6 | 6 | 7 | 6 | 5 | 5 | 6 | 5 | 5 | 5 | 5 | 6 | 5 | 3 | 1 |
|  | Hexp | 0.53 | 0.55 | 0.53 | 0.35 | 0.73 | 0.63 | 0.68 | 0.67 | 0.70 | 0.54 | 0.66 | 0.62 | 0.53 | 0.67 | 0.57 | 0.66 | 0.64 | 0.44 | 0.00 |
|  | Hobs | 0.43 | 0.43 | 0.52 | 0.42 | 0.70 | 0.48 | 0.65 | 0.82 | 0.71 | 0.46 | 0.65 | 0.69 | 0.56 | 0.70 | 0.52 | 0.63 | 0.67 | 0.32 | 0.00 |
|  | Fis | 0.19 | 0.22 | 0.03 | -0.20 | 0.04 | 0.24 | 0.05 | -0.23 | -0.01 | 0.16 | 0.03 | -0.10 | -0.07 | -0.04 | 0.09 | 0.03 | -0.05 | 0.28 | ------ |
| C30 | N | 7 | 7 | 12 | 4 | 2 | 3 | 2 | 2 | 2 | 4 | 2 | 2 | 2 | 3 | 2 | 2 | 2 | 1 | 2 |
|  | Hexp | 0.70 | 0.77 | 0.73 | 0.55 | 0.13 | 0.19 | 0.06 | 0.06 | 0.04 | 0.54 | 0.18 | 0.15 | 0.17 | 0.12 | 0.28 | 0.31 | 0.19 | 0.00 | 0.33 |
|  | Hobs | 0.60 | 0.46 | 0.66 | 0.52 | 0.13 | 0.21 | 0.06 | 0.06 | 0.04 | 0.46 | 0.19 | 0.16 | 0.13 | 0.13 | 0.33 | 0.17 | 0.14 | 0.00 | 0.28 |
|  | Fis | 0.14 | 0.41 | 0.11 | 0.05 | -0.06 | -0.08 | -0.02 | -0.02 | 0.00 | 0.16 | -0.09 | -0.07 | 0.28 | -0.04 | -0.18 | 0.45 | 0.27 | ------ | 0.18 |
| C40 | N | 8 | 3 | 8 | 4 | 4 | 4 | 4 | 6 | 4 | 4 | 5 | 3 | 3 | 4 | 3 | 3 | 3 | 3 | 3 |
|  | Hexp | 0.74 | 0.49 | 0.70 | 0.29 | 0.66 | 0.53 | 0.60 | 0.57 | 0.66 | 0.50 | 0.69 | 0.65 | 0.64 | 0.41 | 0.50 | 0.57 | 0.52 | 0.58 | 0.53 |
|  | Hobs | 0.74 | 0.31 | 0.72 | 0.32 | 0.50 | 0.66 | 0.55 | 0.58 | 0.44 | 0.54 | 0.81 | 0.56 | 0.53 | 0.33 | 0.67 | 0.66 | 0.62 | 0.75 | 0.57 |
|  | Fis | 0.00 | 0.37 | -0.03 | -0.10 | 0.24 | -0.24 | 0.09 | -0.01 | 0.34 | -0.08 | -0.18 | 0.14 | 0.17 | 0.19 | -0.34 | -0.15 | -0.20 | -0.30 | -0.09 |
| S14 | N | 14 | 10 | 13 | 6 | 7 | 9 | 9 | 9 | 7 | 8 | 9 | 9 | 6 | 9 | 8 | 10 | 8 | 5 | 5 |
|  | Hexp | 0.83 | 0.74 | 0.87 | 0.80 | 0.72 | 0.83 | 0.86 | 0.85 | 0.86 | 0.86 | 0.80 | 0.87 | 0.63 | 0.82 | 0.77 | 0.87 | 0.85 | 0.60 | 0.66 |
|  | Hobs | 0.72 | 0.56 | 0.28 | 0.58 | 0.16 | 0.54 | 0.72 | 0.69 | 0.38 | 0.54 | 0.76 | 0.59 | 0.53 | 0.56 | 0.50 | 0.70 | 0.69 | 0.65 | 0.41 |
|  | Fis | 0.14 | 0.25 | 0.69 | 0.29 | 0.78 | 0.36 | 0.16 | 0.19 | 0.57 | 0.38 | 0.06 | 0.32 | 0.16 | 0.32 | 0.36 | 0.20 | 0.19 | -0.10 | 0.38 |
| MIC56 | N | 14 | 14 | 13 | 7 | 4 | 9 | 9 | 11 | 6 | 12 | 13 | 15 | 4 | 9 | 7 | 8 | 12 | 6 | 6 |
|  | Hexp | 0.85 | 0.80 | 0.83 | 0.75 | 0.73 | 0.82 | 0.74 | 0.79 | 0.82 | 0.86 | 0.89 | 0.83 | 0.70 | 0.84 | 0.74 | 0.83 | 0.84 | 0.78 | 0.69 |
|  | Hobs | 0.80 | 0.77 | 0.62 | 0.69 | 0.13 | 0.46 | 0.47 | 0.56 | 0.11 | 0.68 | 0.29 | 0.59 | 0.48 | 0.79 | 0.52 | 0.48 | 0.62 | 0.60 | 0.81 |
|  | Fis | 0.07 | 0.04 | 0.26 | 0.08 | 0.82 | 0.44 | 0.37 | 0.30 | 0.87 | 0.22 | 0.68 | 0.29 | 0.32 | 0.06 | 0.30 | 0.42 | 0.26 | 0.24 | -0.18 |
| EVER07 | N | 5 | 6 | 5 | 3 | 2 | 4 | 3 | 3 | 2 | 4 | 3 | 4 | 2 | 4 | 3 | 3 | 3 | 2 | 1 |
|  | Hexp | 0.65 | 0.53 | 0.65 | 0.13 | 0.10 | 0.29 | 0.11 | 0.12 | 0.13 | 0.29 | 0.09 | 0.15 | 0.09 | 0.32 | 0.16 | 0.42 | 0.07 | 0.04 | 0.00 |
|  | Hobs | 0.58 | 0.60 | 0.69 | 0.13 | 0.10 | 0.32 | 0.11 | 0.12 | 0.13 | 0.32 | 0.10 | 0.16 | 0.09 | 0.34 | 0.17 | 0.34 | 0.07 | 0.04 | 0.00 |
|  | Fis | 0.11 | -0.14 | -0.06 | -0.03 | -0.04 | -0.11 | -0.03 | -0.03 | -0.06 | -0.11 | -0.02 | -0.05 | -0.03 | -0.06 | -0.06 | 0.18 | -0.01 | 0.00 | ------ |
| EVER09 | N | 6 | 4 | 7 | 5 | 7 | 8 | 8 | 8 | 6 | 6 | 5 | 6 | 9 | 7 | 7 | 8 | 7 | 3 | 4 |
|  | Hexp | 0.64 | 0.38 | 0.64 | 0.56 | 0.72 | 0.66 | 0.72 | 0.74 | 0.78 | 0.77 | 0.74 | 0.81 | 0.77 | 0.51 | 0.72 | 0.72 | 0.79 | 0.61 | 0.20 |
|  | Hobs | 0.48 | 0.34 | 0.55 | 0.58 | 0.63 | 0.67 | 0.76 | 0.79 | 0.80 | 0.75 | 0.77 | 0.81 | 0.74 | 0.44 | 0.60 | 0.60 | 0.71 | 0.68 | 0.17 |
|  | Fis | 0.25 | 0.10 | 0.14 | -0.04 | 0.12 | -0.02 | -0.06 | -0.06 | -0.03 | 0.03 | -0.04 | -0.01 | 0.03 | 0.14 | 0.17 | 0.17 | 0.09 | -0.12 | 0.16 |

**Table S3 :** above diagonal: Pairwise F_ST_ values between populations of *Eunicella cavolini* in the Mediterranean (significant values in bold). Below diagonal: pairwise unbiased genetic distance of Nei (1972).

|  | KIA | SPI | DDC | MEN | REV | 3PP | CAV | MEJ | MJS | POU | RID | RIS | SAM | SAR | SOM | VED | VES | AYV | SIV |
| --- | --- | --- | --- | --- | --- | --- | --- | --- | --- | --- | --- | --- | --- | --- | --- | --- | --- | --- | --- |
| KIA |  | **0.02** | 0.00 | **0.14** | **0.10** | **0.18** | **0.17** | **0.18** | **0.16** | **0.17** | **0.16** | **0.15** | **0.22** | **0.17** | **0.16** | **0.12** | **0.14** | **0.20** | **0.27** |
| SPI | 0.04 |  | **0.02** | **0.21** | **0.17** | **0.26** | **0.24** | **0.26** | **0.23** | **0.25** | **0.24** | **0.23** | **0.30** | **0.24** | **0.24** | **0.20** | **0.22** | **0.29** | **0.34** |
| DDC | 0.00 | 0.04 |  | **0.15** | **0.10** | **0.17** | **0.16** | **0.16** | **0.14** | **0.16** | **0.16** | **0.15** | **0.21** | **0.15** | **0.16** | **0.11** | **0.13** | **0.21** | **0.28** |
| MEN | 0.25 | 0.38 | 0.29 |  | **0.04** | **0.21** | **0.14** | **0.16** | **0.14** | **0.17** | **0.14** | **0.13** | **0.18** | **0.17** | **0.12** | **0.14** | **0.10** | **0.28** | **0.31** |
| REV | 0.43 | 0.64 | 0.41 | 0.33 |  | **0.10** | **0.06** | **0.06** | **0.05** | **0.07** | **0.06** | **0.05** | **0.09** | **0.07** | **0.05** | **0.05** | **0.03** | **0.19** | **0.25** |
| 3PP | 0.44 | 0.61 | 0.41 | 0.21 | 0.13 |  | **0.08** | **0.06** | **0.07** | 0.01 | **0.07** | **0.05** | **0.12** | **0.10** | **0.07** | **0.05** | **0.05** | **0.21** | **0.33** |
| CAV | 0.43 | 0.62 | 0.37 | 0.22 | 0.09 | 0.03 |  | **0.02** | **0.02** | **0.03** | **0.03** | **0.03** | **0.05** | 0.01 | **0.02** | **0.02** | **0.03** | **0.24** | **0.32** |
| MEJ | 0.34 | 0.51 | 0.29 | 0.20 | 0.09 | 0.03 | 0.00 |  | 0.00 | **0.02** | **0.03** | **0.02** | **0.05** | **0.04** | **0.02** | **0.03** | **0.03** | **0.23** | **0.32** |
| MJS | 0.43 | 0.65 | 0.40 | 0.26 | 0.03 | 0.05 | 0.03 | 0.03 |  | **0.02** | **0.04** | **0.02** | **0.07** | **0.04** | **0.04** | **0.03** | **0.02** | **0.21** | **0.29** |
| POU | 0.25 | 0.41 | 0.26 | 0.05 | 0.18 | 0.10 | 0.10 | 0.08 | 0.13 |  | 0.00 | 0.00 | **0.06** | **0.07** | **0.03** | 0.01 | 0.01 | **0.16** | **0.29** |
| RID | 0.40 | 0.64 | 0.40 | 0.20 | 0.11 | 0.05 | 0.05 | 0.05 | 0.02 | 0.10 |  | 0.00 | **0.03** | **0.05** | **0.03** | **0.03** | **0.02** | **0.18** | **0.28** |
| RIS | 0.38 | 0.58 | 0.37 | 0.20 | 0.07 | 0.05 | 0.02 | 0.03 | 0.01 | 0.08 | 0.01 |  | **0.05** | **0.06** | **0.03** | **0.02** | **0.02** | **0.17** | **0.26** |
| SAM | 0.52 | 0.76 | 0.50 | 0.25 | 0.16 | 0.06 | 0.06 | 0.09 | 0.08 | 0.13 | 0.04 | 0.07 |  | **0.06** | **0.07** | **0.06** | **0.07** | **0.26** | **0.33** |
| SAR | 0.38 | 0.53 | 0.32 | 0.24 | 0.15 | 0.02 | 0.05 | 0.06 | 0.10 | 0.11 | 0.07 | 0.09 | 0.08 |  | **0.04** | **0.03** | **0.03** | **0.27** | **0.36** |
| SOM | 0.36 | 0.53 | 0.36 | 0.16 | 0.10 | 0.03 | 0.03 | 0.05 | 0.04 | 0.07 | 0.05 | 0.04 | 0.08 | 0.05 |  | **0.02** | **0.02** | **0.21** | **0.31** |
| VED | 0.32 | 0.52 | 0.30 | 0.22 | 0.09 | 0.03 | 0.06 | 0.05 | 0.04 | 0.10 | 0.06 | 0.06 | 0.07 | 0.04 | 0.05 |  | 0.01 | **0.19** | **0.28** |
| VES | 0.32 | 0.50 | 0.30 | 0.14 | 0.07 | 0.04 | 0.04 | 0.03 | 0.02 | 0.06 | 0.03 | 0.03 | 0.09 | 0.05 | 0.02 | 0.03 |  | **0.18** | **0.27** |
| AYV | 0.36 | 0.57 | 0.42 | 0.42 | 0.30 | 0.38 | 0.35 | 0.29 | 0.22 | 0.31 | 0.26 | 0.24 | 0.38 | 0.42 | 0.30 | 0.28 | 0.25 |  | **0.24** |
| SIV | 0.44 | 0.62 | 0.51 | 0.38 | 0.48 | 0.48 | 0.45 | 0.39 | 0.37 | 0.37 | 0.40 | 0.36 | 0.46 | 0.56 | 0.42 | 0.42 | 0.37 | 0.23 |  |

**Table S4 :** Pairwise FST values between populations of *Eunicella cavolini* in the Mediterranean using the ENA correction described in Chapuis and Estoup (2007).

| **pop** | **KIA** | **SPI** | **DDC** | **MEN** | **3PP** | **CAV** | **MEJ** | **MJS** | **POU** | **REV** | **RID** | **RIS** | **SAM** | **SAR** | **SOM** | **VED** | **VES** | **AYV** |
| --- | --- | --- | --- | --- | --- | --- | --- | --- | --- | --- | --- | --- | --- | --- | --- | --- | --- | --- |
| **SPI** | 0.02 |  |  |  |  |  |  |  |  |  |  |  |  |  |  |  |  |  |
| **DDC** | 0.00 | 0.02 |  |  |  |  |  |  |  |  |  |  |  |  |  |  |  |  |
| **MEN** | 0.14 | 0.21 | 0.16 |  |  |  |  |  |  |  |  |  |  |  |  |  |  |  |
| **3PP** | 0.17 | 0.27 | 0.18 | 0.21 |  |  |  |  |  |  |  |  |  |  |  |  |  |  |
| **CAV** | 0.17 | 0.24 | 0.16 | 0.14 | 0.07 |  |  |  |  |  |  |  |  |  |  |  |  |  |
| **MEJ** | 0.18 | 0.26 | 0.17 | 0.16 | 0.05 | 0.01 |  |  |  |  |  |  |  |  |  |  |  |  |
| **MJS** | 0.15 | 0.23 | 0.14 | 0.14 | 0.05 | 0.02 | 0.00 |  |  |  |  |  |  |  |  |  |  |  |
| **POU** | 0.18 | 0.28 | 0.18 | 0.19 | 0.01 | 0.03 | 0.02 | 0.02 |  |  |  |  |  |  |  |  |  |  |
| **REV** | 0.10 | 0.17 | 0.10 | 0.05 | 0.09 | 0.05 | 0.06 | 0.05 | 0.08 |  |  |  |  |  |  |  |  |  |
| **RID** | 0.16 | 0.25 | 0.16 | 0.15 | 0.06 | 0.03 | 0.03 | 0.04 | 0.01 | 0.06 |  |  |  |  |  |  |  |  |
| **RIS** | 0.15 | 0.24 | 0.16 | 0.14 | 0.04 | 0.03 | 0.02 | 0.02 | 0.01 | 0.05 | 0.01 |  |  |  |  |  |  |  |
| **SAM** | 0.21 | 0.29 | 0.21 | 0.17 | 0.10 | 0.05 | 0.04 | 0.07 | 0.06 | 0.09 | 0.03 | 0.05 |  |  |  |  |  |  |
| **SAR** | 0.17 | 0.25 | 0.16 | 0.17 | 0.10 | 0.01 | 0.03 | 0.04 | 0.07 | 0.07 | 0.05 | 0.06 | 0.06 |  |  |  |  |  |
| **SOM** | 0.16 | 0.24 | 0.16 | 0.11 | 0.06 | 0.02 | 0.02 | 0.03 | 0.03 | 0.04 | 0.03 | 0.03 | 0.06 | 0.03 |  |  |  |  |
| **VED** | 0.11 | 0.19 | 0.11 | 0.13 | 0.04 | 0.02 | 0.03 | 0.03 | 0.03 | 0.04 | 0.04 | 0.03 | 0.06 | 0.04 | 0.02 |  |  |  |
| **VES** | 0.13 | 0.22 | 0.13 | 0.10 | 0.04 | 0.02 | 0.02 | 0.02 | 0.02 | 0.03 | 0.02 | 0.02 | 0.06 | 0.03 | 0.01 | 0.01 |  |  |
| **AYV** | 0.21 | 0.30 | 0.23 | 0.29 | 0.19 | 0.25 | 0.23 | 0.21 | 0.16 | 0.20 | 0.19 | 0.18 | 0.26 | 0.27 | 0.21 | 0.19 | 0.19 |  |
| **SIV** | 0.27 | 0.35 | 0.29 | 0.31 | 0.31 | 0.32 | 0.30 | 0.28 | 0.29 | 0.25 | 0.28 | 0.26 | 0.32 | 0.35 | 0.30 | 0.27 | 0.27 | 0.24 |

**Table S5 :** Results of AMOVA with five groups (by separating the two islands of Menorca and Corsica). The significance of the different parameters was tested with permutations (n = 1000 of each analysis).

| **Source of variation** | **R_ST_ like analysis** | | **F_ST_ like analysis** | |
| --- | --- | --- | --- | --- |
|  | Percentage of variation | Fixation index | Percentage of variation | Fixation index |
| Among groups | 6.08 % | F_CT_ = 0.04 (p= 0.03) | 16.16 % | F_CT_ = 0.19  (p= 0) |
| Among populations within groups | 0% | F_SC_ = -0.01  (p = 0.99) | 2.92 % | F_SC_ = 0.03  (p = 0) |
| Within populations | 95.5 % | F_ST_ = 0.06  (p = 0) | 80.93 % | F_ST_ = 0.16  (p = 0) |

**Table S6 :** proportions of successful reassignment of individuals to their original clusters for the DAPC analysis.

| **1** | **2** | **3** | **4** | **5** | **6** | **7** | **8** |
| --- | --- | --- | --- | --- | --- | --- | --- |
| 0.49 | 0.71 | 0.97 | 0.49 | 0.79 | 0.87 | 0.83 | 0.94 |
| **9** | **10** | **11** | **12** | **13** | **14** | **15** |  |
| 0.90 | 0.60 | 1.00 | 0.96 | 0.88 | 0.72 | 0.73 |  |


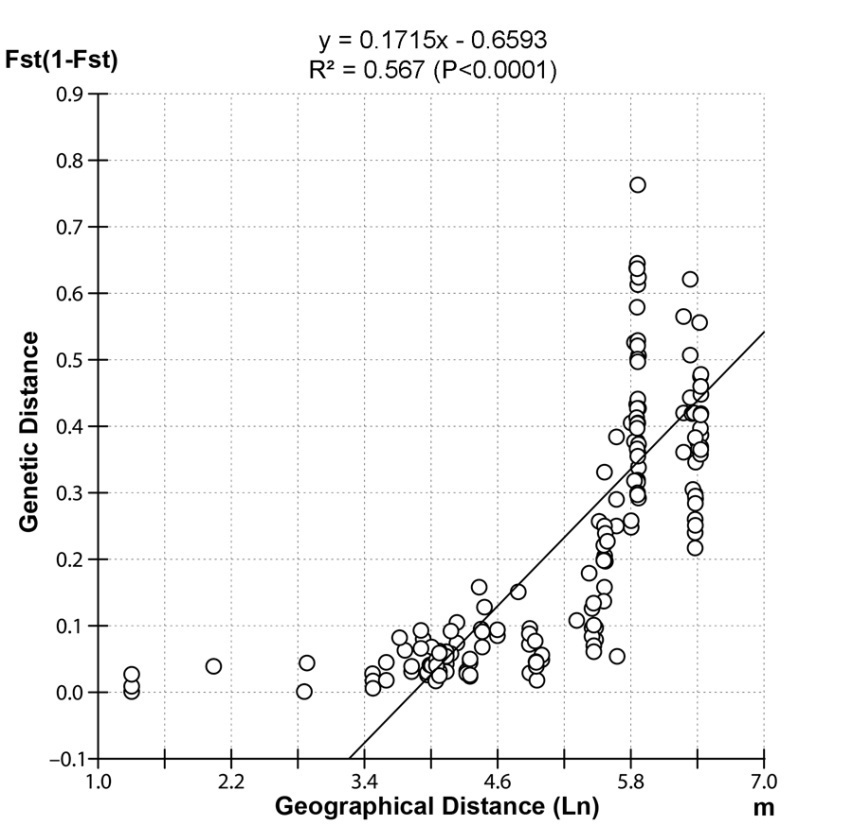


**Figure S1 :** Correlation between genetic (F_ST_/(1-F_ST_)) and logarithm of geographical distances between sample pairs (whole data set).


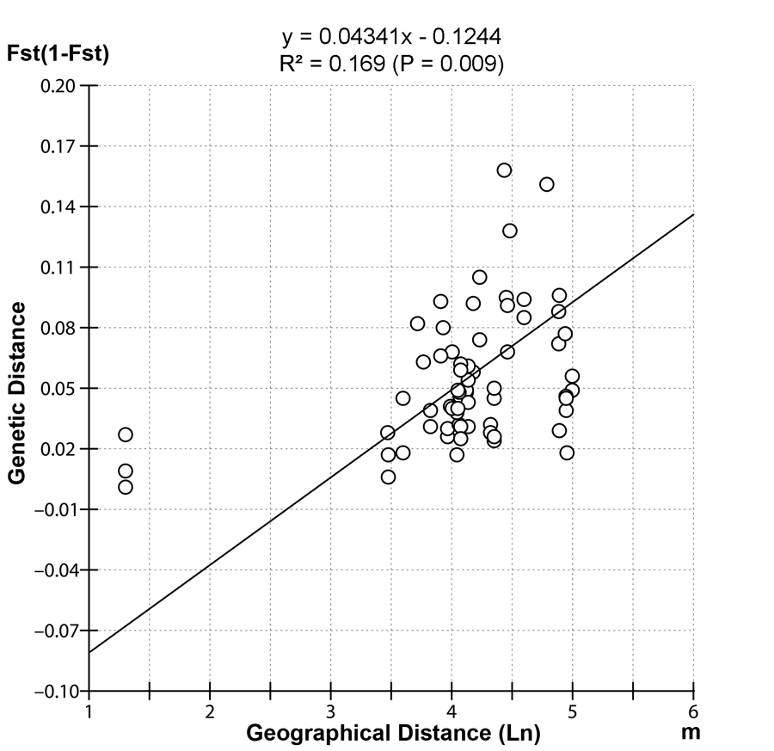


**Figure S2 :** Correlation between genetic (F_ST_/(1-F_ST_)) and logarithm of geographical distances between sample pairs within the French region.


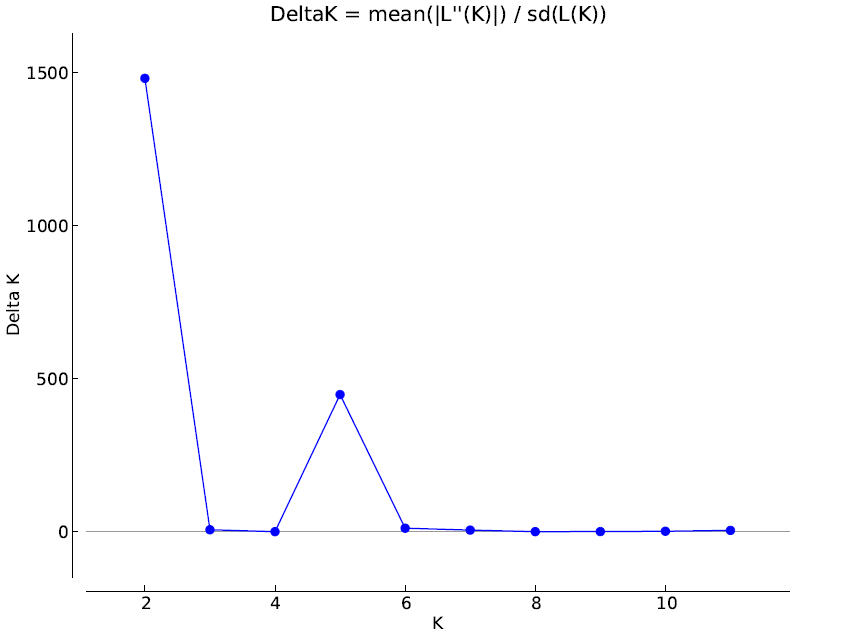


**Figure S3 :** evolution of Delta(K) for the first round of STRUCTURE (whole data set).

.
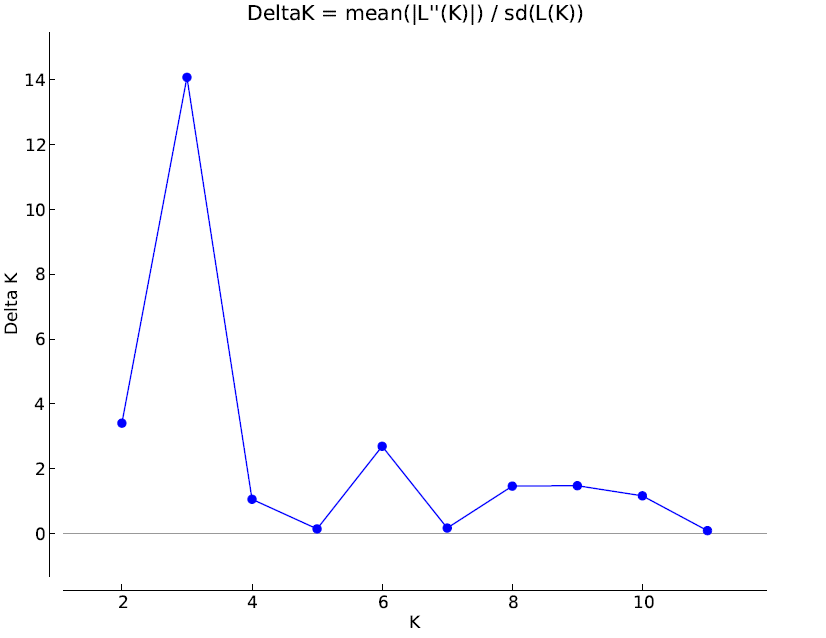


**Figure S4 :** evolution of Delta(K) for the second round of STRUCTURE on French samples alone.


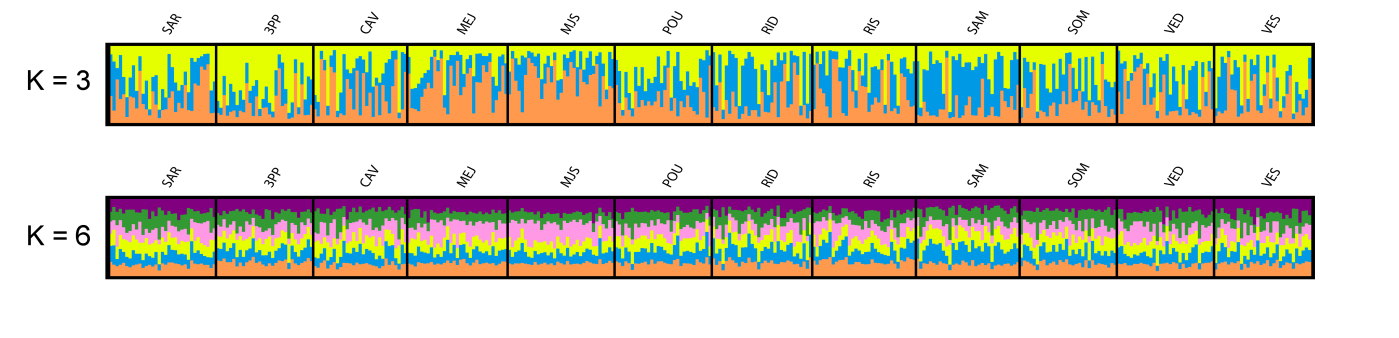


**Figure S5 :** bar plot from the analysis with STRUCTURE on French populations alone.


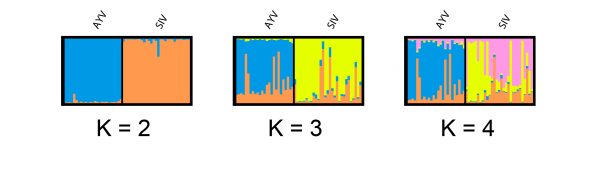


**Figure S6 :** bar plot from the analysis with STRUCTURE on Turkish populations alone.


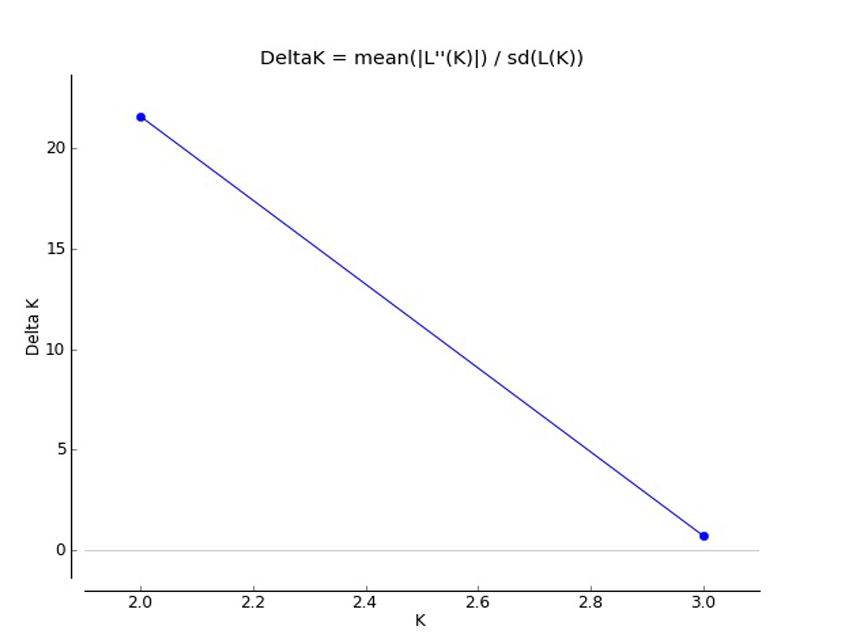


**Figure S7 :** evolution of Delta(K) for STRUCTURE round on Menorca and Balearic islands.


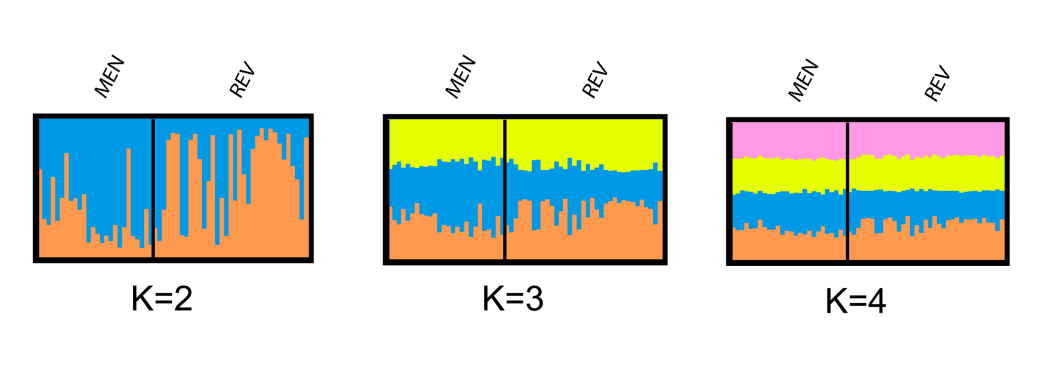


**Figure S8 :** bar plot from the analysis with STRUCTURE on Menorca and Corsica islands alone.


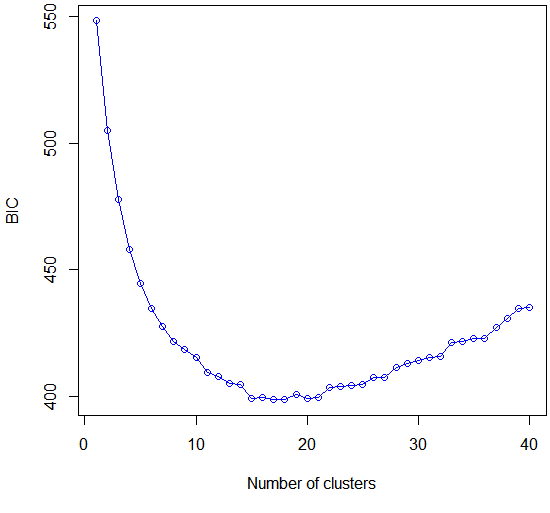


**Figure S9 :** graph of Bayesian Information Criteria (BIC) values for increasing values of K (whole data set).


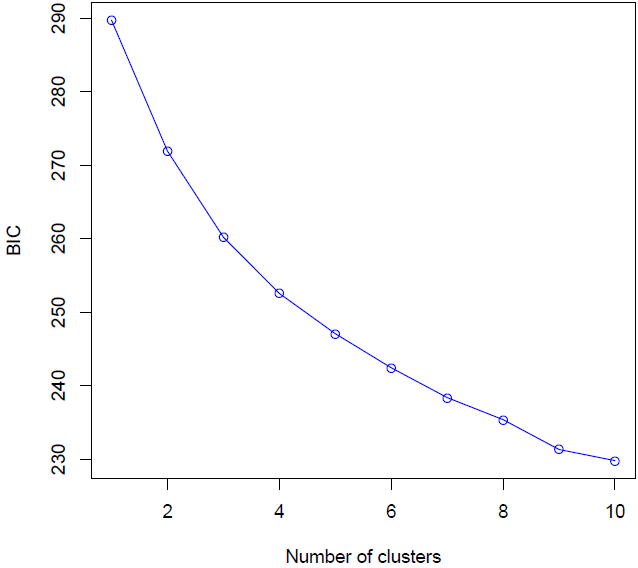


**Figure S10 :** graph of Bayesian Information Criteria (BIC) values for increasing values of K (French data set).


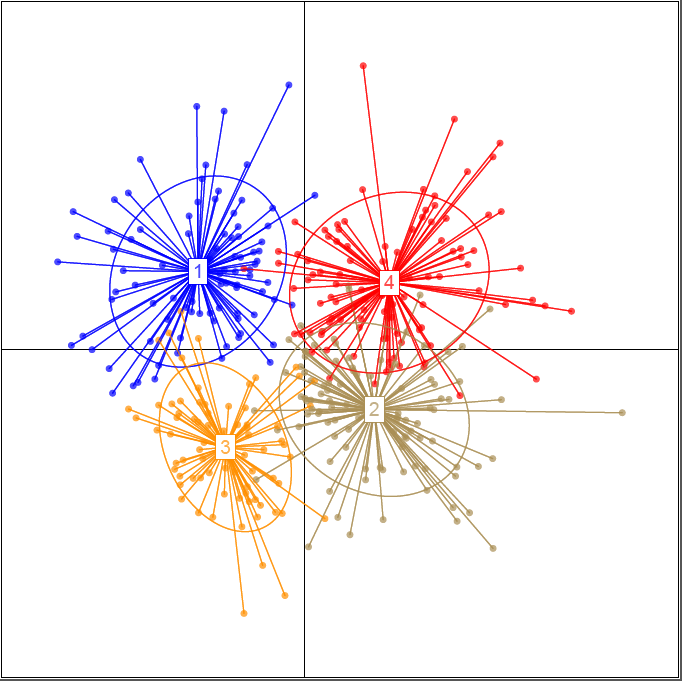


**Figure S11 :** results of the DAPC analysis applied on French samples with K = 4 (second round of DAPC).
