# Supplementary material for: Contrasted levels of genetic diversity in a benthic Mediterranean octocoral: Consequences of different demographic histories?
Source: Ecol Evol. 2016 Oct 28;6(24):8665–78. doi: 10.1002/ece3.2490 (PMC5192949; doi:10.1002/ece3.2490)
Supplement: Supplementary file 3 [file ECE3-6-8665-s003.docx]

**Appendix S3.**

**Table S1 :** MSVAR results: median, mean, variance (Var) and standard deviation (SD) for current and ancestral population size in separate samples. Ratio: N_anc_ / N_curr_. Values are expressed in log_10_.

| Region | **Algeria** | | | |  | **France** | | | |  |
| --- | --- | --- | --- | --- | --- | --- | --- | --- | --- | --- |
| Population | **KIA/DDC** | | **SPI** | |  | **MJS** | | **RIS** | |  |
| Size | **N_curr_** | **N_anc_** | **N_curr_** | **N_anc_** | **Ratio** | **N_curr_** | **N_anc_** | **N_curr_** | **N_anc_** | **Ratio** |
| mean | 2.03 | 5.18 | 1.21 | 5.32 | 4.11 | 1.97 | 5.09 | 1.48 | 5.17 | 3.69 |
| median | 2.02 | 5.19 | 1.23 | 5.33 |  | 1.95 | 5.09 | 1.45 | 5.17 |  |
| SD | 0.51 | 0.46 | 0.69 | 0.49 |  | 0.53 | 0.5 | 0.54 | 0.51 |  |
| Var | 0.26 | 0.21 | 0.47 | 0.24 |  | 0.28 | 0.25 | 0.3 | 0.26 |  |
|  |  |  |  |  |  |  |  |  |  |  |
| Region | **Islands** | | | |  | **Turkey** | | | |  |
| Population | **MEN** | | **REV** | |  | **AYV** | | **SIV** | |  |
| Size | **N_curr_** | **N_anc_** | **N_curr_** | **N_anc_** | **Ratio** | **N_curr_** | **N_anc_** | **N_curr_** | **N_anc_** | **Ratio** |
| mean | 1.19 | 5.27 | 1.15 | 5.3 | 4.15 | 0.78 | 4.89 | 0.67 | 5.06 | 4.39 |
| median | 1.2 | 5.28 | 1.16 | 5.3 |  | 0.8 | 4.9 | 0.72 | 5.07 |  |
| SD | 0.56 | 0.49 | 0.69 | 0.48 |  | 0.79 | 0.59 | 0.73 | 0.6 |  |
| Var | 0.32 | 0.24 | 0.48 | 0.23 |  | 0.62 | 0.35 | 0.54 | 0.36 |  |

**Table S2 :** MSVAR results: median, mean, variance (Var) and standard deviation (SD) for current and ancestral population size in four regions of the Mediterranean. Ratio: N_anc_ / N_curr_. µ: mutation rate, T: time since the population size change. Values are expressed in log_10_.

|  | **Algeria** | | | | |  | **France** | | | | |
| --- | --- | --- | --- | --- | --- | --- | --- | --- | --- | --- | --- |
|  | **N_curr_** | **N_anc_** | **Ratio** | **µ** | **Time** |  | **N_curr_** | **N_anc_** | **Ratio** | **µ** | **Time** |
| Mean | 1.94 | 5.21 | 3.27 | -3.21 | 2.98 |  | 1.32 | 5.27 | 3.95 | -3.29 | 2.70 |
| Median | 1.93 | 5.21 |  | -3.21 | 2.98 |  | 1.33 | 5.28 |  | -3.30 | 2.71 |
| SD | 0.54 | 0.46 |  | 0.44 | 0.52 |  | 0.58 | 0.48 |  | 0.45 | 0.56 |
| Var | 0.29 | 0.21 |  | 0.20 | 0.27 |  | 0.33 | 0.23 |  | 0.20 | 0.31 |
|  |  | | | | | | | | | | |
|  | **Islands** | | | | |  | **Turkey** | | | | |
|  | **N_curr_** | **N_anc_** | **Ratio** | **µ** | **Time** |  | **N_curr_** | **N_anc_** | **Ratio** | **µ** | **Time** |
| Mean | 1.27 | 5.28 | 4.01 | -3.30 | 2.68 |  | 1.10 | 4.92 | 3.81 | -3.35 | 2.72 |
| Median | 1.28 | 5.29 |  | -3.30 | 2.69 |  | 1.13 | 4.93 |  | -3.35 | 2.74 |
| SD | 0.56 | 0.47 |  | 0.45 | 0.54 |  | 0.65 | 0.53 |  | 0.45 | 0.61 |
| Var | 0.32 | 0.22 |  | 0.20 | 0.29 |  | 0.43 | 0.28 |  | 0.20 | 0.37 |

**Table S3 :** MSVAR results: median, mean, variance (Var) and standard deviation (SD) for current and ancestral population size in the French area with a pool of 24 individuals, two for each sample.

|  | **N_curr_** | **N_anc_** | **Ratio** |
| --- | --- | --- | --- |
| mean | 1.61 | 4.04 | 2.43 |
| median | 1.64 | 4.04 |  |
| SD | 0.80 | 0.59 |  |
| Var | 0.65 | 0.35 |  |

**Table S4 :** MSVAR results: median, mean, variance (Var) and standard deviation (SD) for current and ancestral population size for each of the seven loci in the Algerian region.

| **Loci** | **C21** | | **C30** | | **C40** | | **S14** | | **Mic56** | | **Ever007** | | **Ever009** | |
| --- | --- | --- | --- | --- | --- | --- | --- | --- | --- | --- | --- | --- | --- | --- |
| **Size** | **N_curr_** | **N_anc_** | **N_curr_** | **N_anc_** | **N_curr_** | **N_anc_** | **N_curr_** | **N_anc_** | **N_curr_** | **N_anc_** | **N_curr_** | **N_anc_** | **N_curr_** | **N_anc_** |
| **mean** | 1.55 | 5.19 | 1.91 | 5.26 | 1.9 | 5.21 | 2.02 | 5.26 | 2.03 | 5.26 | 1.72 | 5.18 | 1.73 | 5.22 |
| **median** | 1.53 | 5.19 | 1.9 | 5.27 | 1.89 | 5.21 | 2.02 | 5.25 | 2.04 | 5.26 | 1.71 | 5.18 | 1.72 | 5.22 |
| **SD** | 0.58 | 0.48 | 0.58 | 0.47 | 0.59 | 0.47 | 0.59 | 0.46 | 0.59 | 0.46 | 0.58 | 0.48 | 0.57 | 0.47 |
| **Var** | 0.34 | 0.23 | 0.34 | 0.22 | 0.35 | 0.22 | 0.35 | 0.21 | 0.35 | 0.21 | 0.34 | 0.23 | 0.33 | 0.22 |


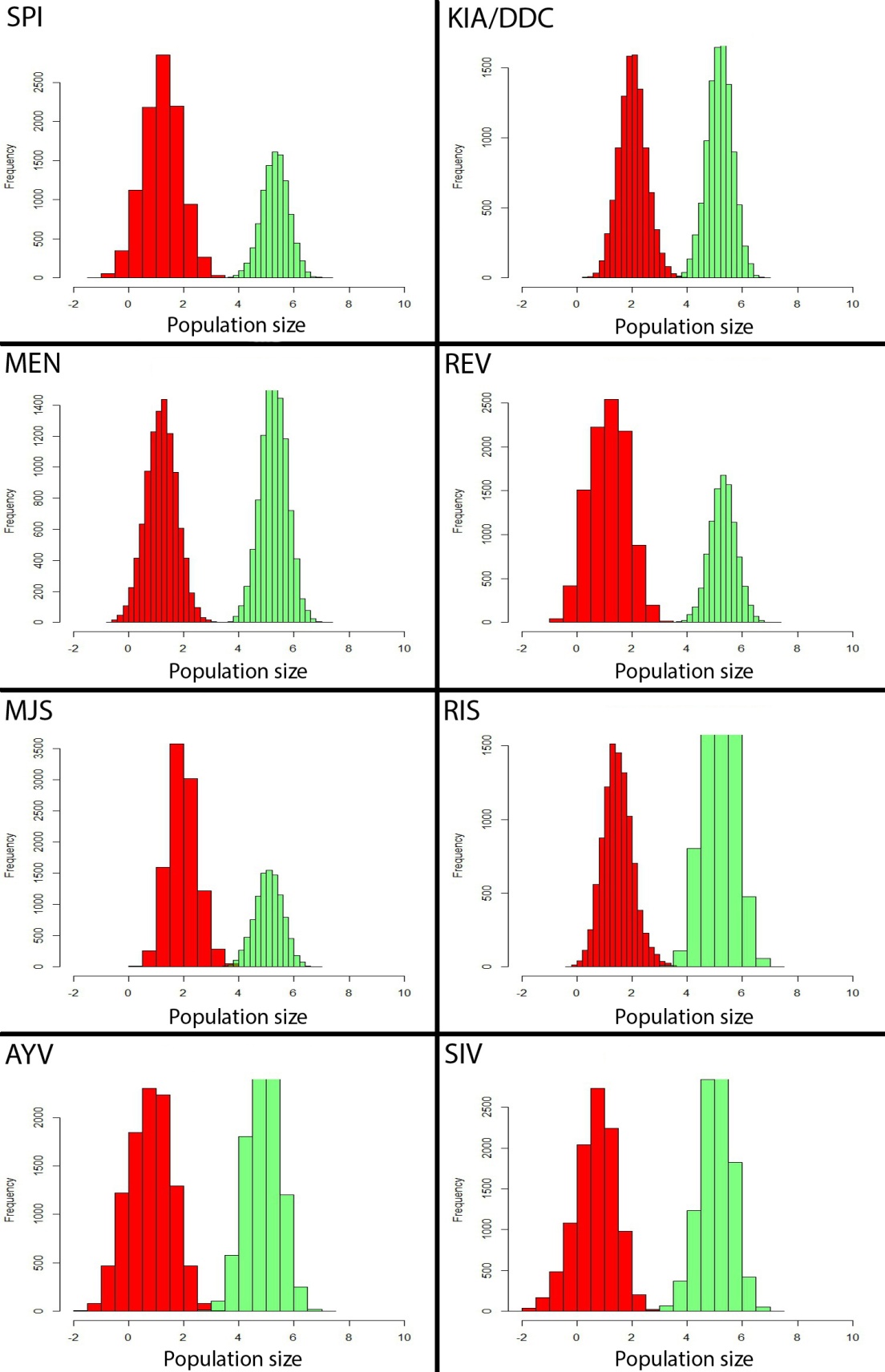


**Figure S1 :** Results of MSVAR analysis: marginal posterior density of current (red) and ancestral (green) effective population size in eight populations separately. Densities are expressed in a log_10_ scale. See main text for population codes.


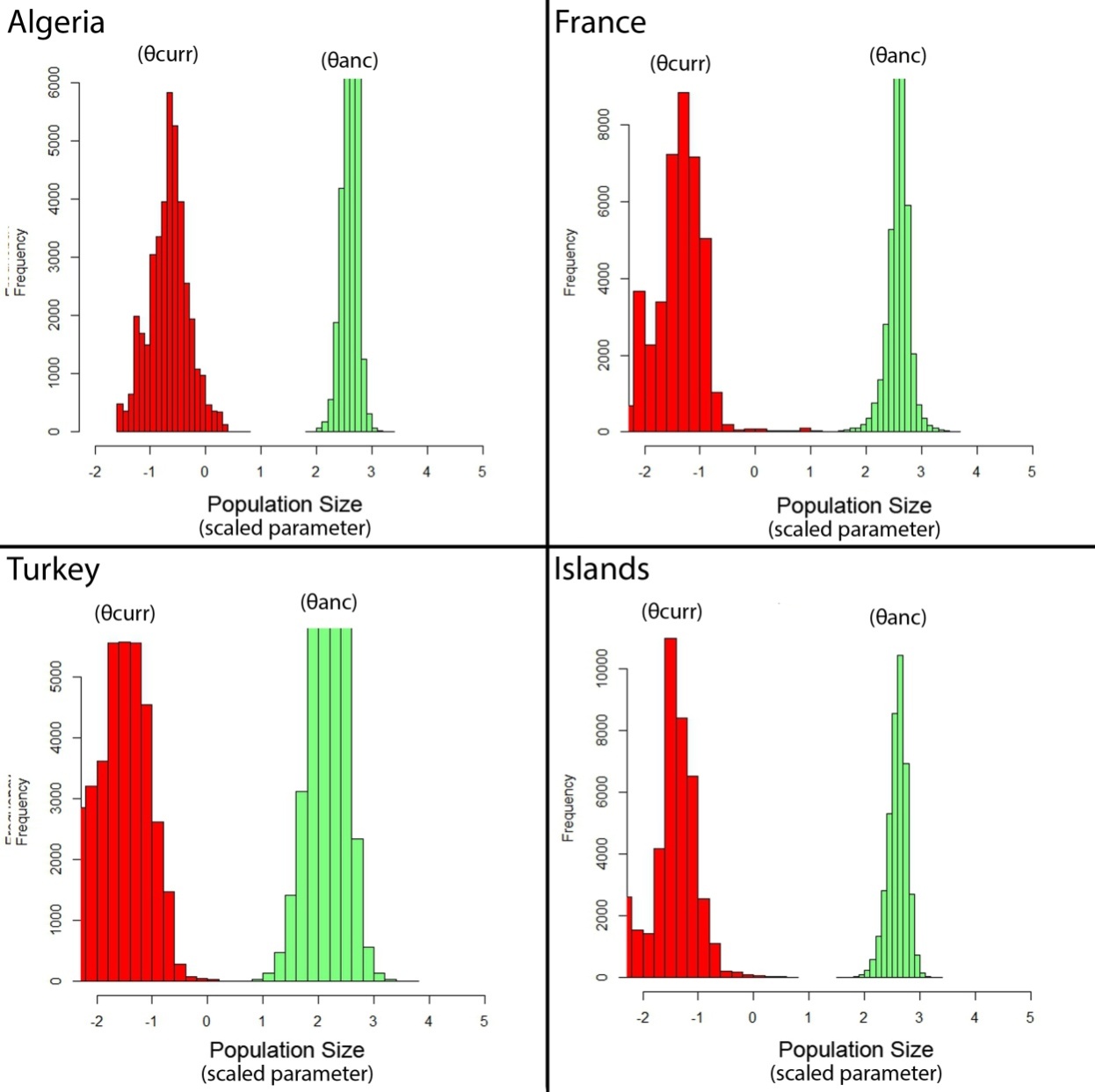


**Figure S2 :** Results of MSVAR analysis: marginal posterior density of scaled parameters. Current (red) and ancestral (green) effective population size in four regions of the Mediterranean. Densities are expressed in a log_10_ scale.


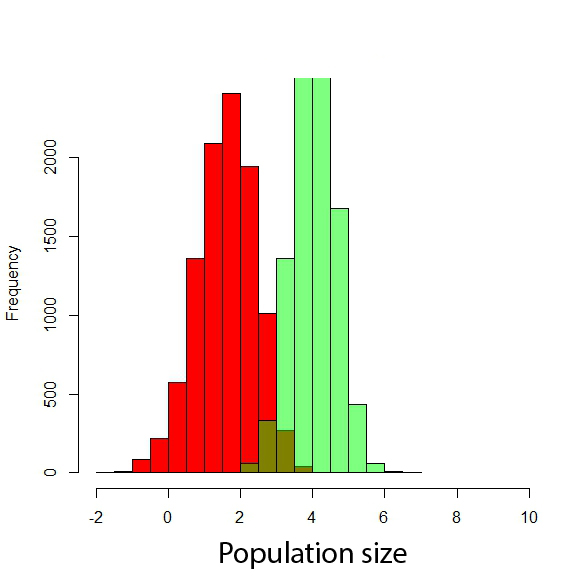


**Figure S3 :** Results of MSVAR analysis: marginal posterior density of current (red) and ancestral (green) effective population size over seven loci in the French region with 24 individuals, two for each sample. Densities are expressed in a log_10_ scale.


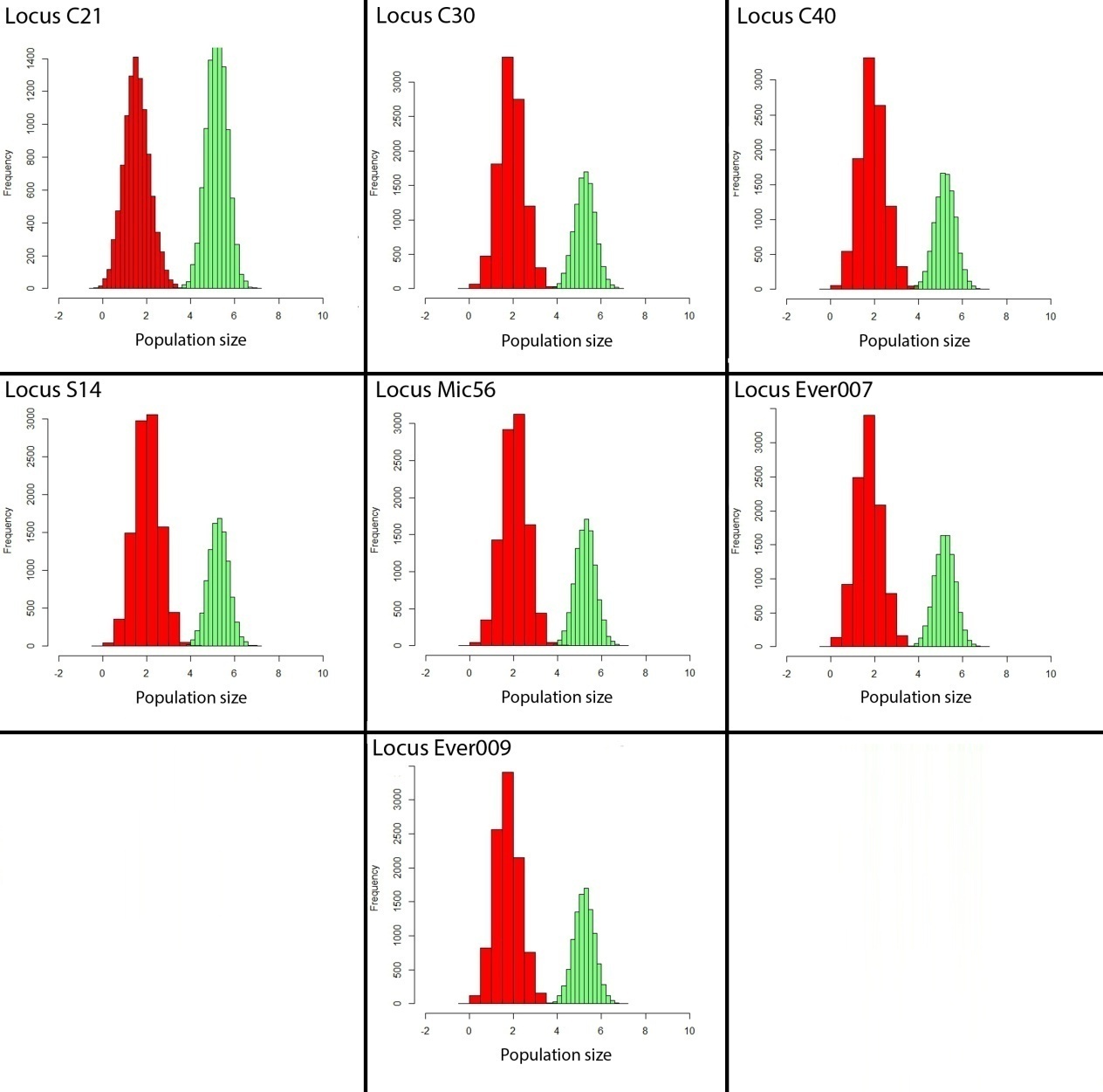


**Figure S4 :** Results of MSVAR analysis: marginal posterior density of current (red) and ancestral (green) effective population size over seven loci in the Algerian region. Densities are expressed in a log_10_ scale.
